# Budesonide/formoterol fumarate dihydrate *versus* budesonide with a novel suspension technology for uncontrolled asthma: phase 3 VATHOS study

**DOI:** 10.1183/23120541.01817-2025

**Published:** 2026-09-14

**Authors:** David J. Jackson, Robert A. Wise, Alberto Papi, Njira Lugogo, Ruchong Chen, Hassan Chami, Clara Armengol, Sami Z. Daoud, Emma Nauclér, Karin Bowen, Ayman Megally, Olami Sobande, Mehul Patel

**Affiliations:** 1 Guy's Severe Asthma Centre, Guy's Hospital, School of Immunology and Microbial Sciences, King's College London, London, UK; 2 Department of Medicine, Johns Hopkins University School of Medicine, Baltimore, MD, USA; 3 Respiratory Medicine, Department of Translational Medicine, University of Ferrara, Ferrara, Italy; 4 Division of Pulmonary and Critical Care Medicine, University of Michigan, Ann Arbor, MI, USA; 5 State Key Laboratory of Respiratory Disease, Joint International Research Laboratory of Respiratory Health, National Clinical Research Center for Respiratory Disease, National Center for Respiratory Medicine, Department of Allergy and Clinical Immunology, Guangzhou Institute of Respiratory Health, The First Affiliated Hospital of Guangzhou Medical University, Guangzhou, PR China; 6 Guangzhou National Laboratory, Guangzhou, PR China; 7 Respiratory and Immunology, Clinical Development, Biopharmaceuticals R&D, AstraZeneca, Gaithersburg, MD, USA; 8 Respiratory and Immunology, Clinical Development, Biopharmaceuticals R&D, AstraZeneca, Barcelona, Spain; 9 Respiratory and Immunology, Biometrics, Biopharmaceuticals R&D, AstraZeneca, Gothenburg, Sweden; 10 Respiratory and Immunology, Biometrics, Biopharmaceuticals R&D, AstraZeneca, Gaithersburg, MD, USA; 11 Respiratory and Immunology, Clinical Development, Biopharmaceuticals R&D, AstraZeneca, Cambridge, UK

## Abstract

**Background:**

Inhaled corticosteroid (ICS)/long-acting β_2_-agonist (LABA) therapy is important in asthma. The phase 3 VATHOS study (ClinicalTrials.gov: NCT05202262) assessed the efficacy and safety of budesonide/formoterol fumarate dihydrate (BFF) *via* metered-dose inhaler (MDI) using Aerosphere co-suspension technology (BFF_A_) *versus* either budesonide (BD) *via* MDI using Aerosphere or BFF *via* dry-powder inhaler (BFF_DPI_).

**Methods:**

Participants (age 12–80 years; n=645) with inadequately controlled asthma on medium-dose ICS or ICS/LABA were randomised 1:2:2:2 to twice-daily BFF 160/10 μg (BFF_A_ 160), BFF 320/10 μg (BFF_A_ 320), BD 320 μg (BD 320) or open-label BFF_DPI_ 320/9 μg, respectively. End-points included change from baseline in forced expiratory volume in 1 s area under the curve from 0 to 3 h (FEV_1_ AUC_0–3_) at 24 weeks (primary), and morning pre-dose trough FEV_1_ at 24 weeks, onset of action (day 1 change in FEV_1_, 5 min post-dose) and severe exacerbation rates (secondary). Adverse events (AEs) were recorded.

**Results:**

BFF_A_ 320 demonstrated superior lung function *versus* BD 320 at week 24 (mean change from baseline in FEV_1_ AUC_0–3_: 173 (95% CI 112–233) mL (p<0.0001); trough FEV_1_: 61 (95% CI 1–120) mL (p=0.0447); onset of action: 144 (95% CI 110–177) mL (p<0.0001)). Severe exacerbation rates were similar between BFF_A_ 320 and BD 320 (rate ratio 1.02 (95% CI 0.61–1.70); p=0.9473). AEs were similar across treatments (BFF_A_ 320, 50.3%; BFF_A_ 160, 50.0%; BD 320, 38.1%; BFF_DPI_, 45.7%). BFF_A_ 320 showed non-inferiority to BFF_DPI_ in lung function.

**Conclusion:**

These findings support the use of BFF_A_ 320 with co-suspension delivery technology in people with inadequately controlled asthma on medium-dose ICS or ICS/LABA.

## Shareable abstract

**Using Aerosphere co-suspension MDI technology, single-inhaler budesonide/formoterol improved lung function and enhanced onset of action over 5 min post-dose *versus* budesonide in people with inadequately controlled asthma** https://bit.ly/4ojuBIF

## Introduction

Asthma is a heterogeneous disease characterised by chronic airway inflammation, respiratory symptoms and variable levels of expiratory airflow limitation [1]. The goals of asthma management include achieving symptom control, minimising the risk of asthma exacerbations, asthma-related mortality, progressive airflow limitation and adverse events (AEs) associated with treatment. Combined inhaled corticosteroids (ICS) and long-acting β_2_-agonists (LABA) are the mainstay treatment for asthma [1–3], and have demonstrated greater improvements in lung function and exacerbation rates in adolescents and adults with asthma *versus* ICS alone [4–6]. Current recommendations from the Global Initiative for Asthma for moderate-to-severe asthma maintenance therapy in adults and adolescents aged ≥12 years emphasise a personalised, control-based, stepwise approach to maintenance therapy, in which treatment is reviewed and adjusted based on ongoing assessment. Initial treatment typically includes low-dose ICS-containing therapy, preferably with LABA as a MART (maintenance and reliever therapy), with escalation to regular maintenance medium-dose ICS/LABA if asthma remains uncontrolled [1].

Co-suspension delivery technology (Aerosphere) *via* metered-dose inhaler (MDI) comprises spray-dried porous particles with the phospholipid 1,2-distearoyl-*sn*-glycero-3-phosphocholine and calcium chloride in a propellant [7, 8]. Studies using functional respiratory imaging have found that these particles form strong non-specific associations with the active pharmaceutical agents, providing long-term stability and consistent, balanced drug delivery throughout the large and small airways [9]. Budesonide/glycopyrronium/formoterol fumarate dihydrate triple therapy delivered using Aerosphere is already marketed for maintenance treatment in people with COPD in over 50 countries worldwide, including the USA and European Union [10–12]. While approved dual ICS/LABA MDIs are important therapeutic options in asthma, an ICS/LABA Aerosphere co-suspension formulation represents another potential treatment option for people with asthma who do not achieve adequate disease control with currently available therapies.

Budesonide/formoterol fumarate dihydrate (BFF), delivered *via* MDI using the same co-suspension delivery technology, is now being evaluated in two phase 3 clinical trials in asthma: LITHOS (ClinicalTrials.gov: NCT05755906) and VATHOS (ClinicalTrials.gov: NCT05202262). The 24-week VATHOS study described here evaluated the safety and superiority of medium-dose BFF *via* MDI using Aerosphere co-suspension delivery technology (BFF_A_) on lung function (including onset of action) and asthma health-related quality of life *versus* budesonide (BD) *via* MDI also using Aerosphere co-suspension technology, in participants aged 12–80 years with inadequately controlled asthma, despite treatment with medium-dose ICS or ICS/LABA. We also performed prespecified and *post hoc* analyses comparing BFF_A_ *via* dry-powder inhaler (BFF_DPI_) as a benchmark for practising clinicians. Additionally, we report severe exacerbation rates pooled from VATHOS and LITHOS data; LITHOS differs from VATHOS in that it included only two treatment arms (lower dose BFF and BD) and was of shorter duration (12 weeks).

## Methods

### Design and treatment

**FIGURE 1:**
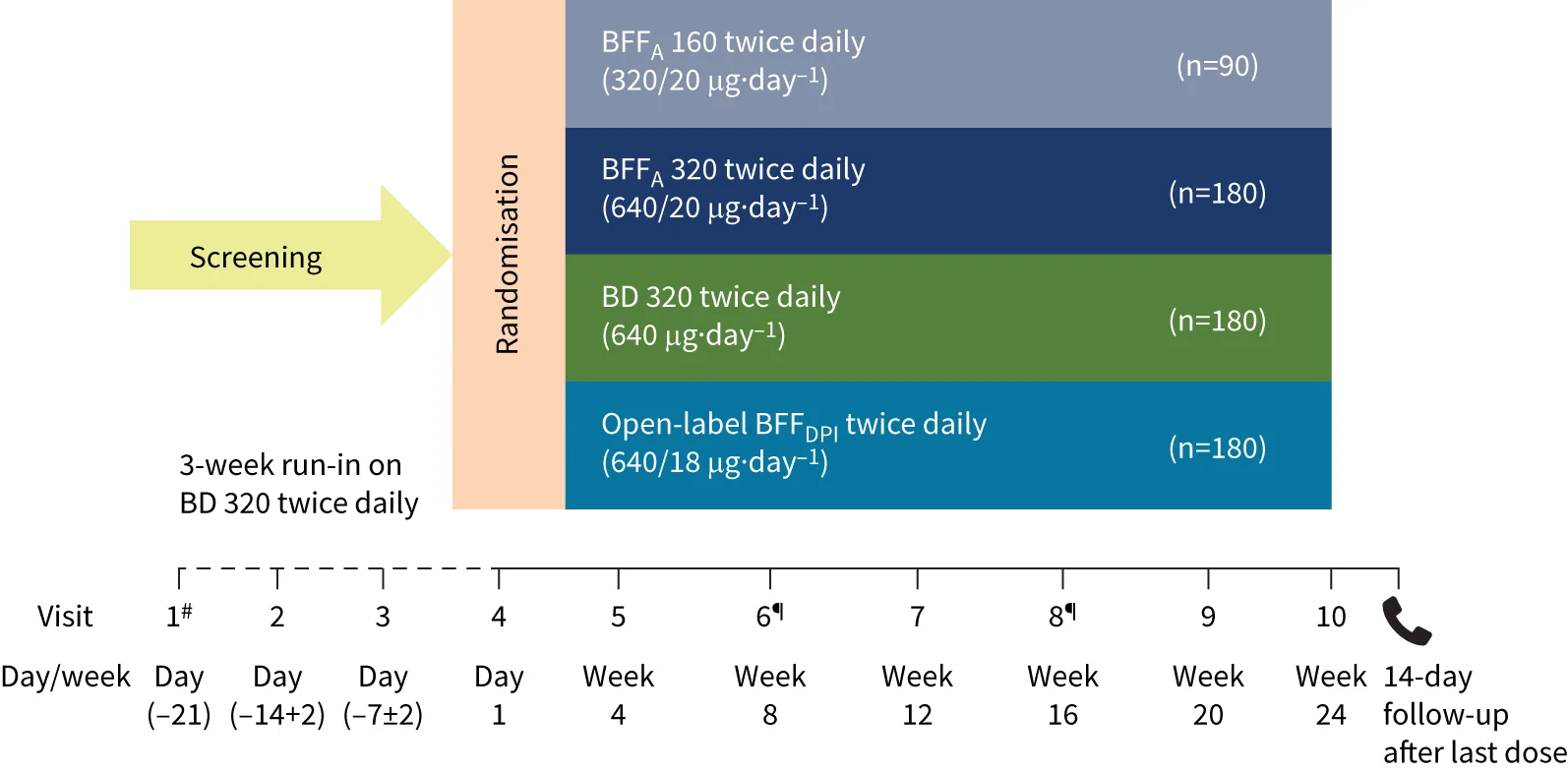
Study design schematic showing target randomisation numbers. ^#^: stop daily ICS/LABA or ICS regimen and start run-in with BD 320 twice daily; ^¶^: telemedicine visit (phone or video). BD 320: budesonide 320 μg *via* Aerosphere MDI; BFF_A_ 160: budesonide/formoterol fumarate dihydrate 160/10 μg *via* Aerosphere MDI; BFF_A_ 320: budesonide/formoterol fumarate dihydrate 320/10 μg *via* Aerosphere MDI; BFF_DPI_: budesonide/formoterol fumarate dihydrate 320/9 μg *via* dry-powder inhaler; ICS: inhaled corticosteroid; LABA: long-acting β_2_-agonist; MDI: metered-dose inhaler.

VATHOS is a 24-week, phase 3, randomised, double-blind, parallel group, multicentre study (figure 1) in adults and adolescents with inadequately controlled asthma (7-item Asthma Control Questionnaire (ACQ-7) total score ≥1.5) despite treatment with medium-dose ICS or ICS/LABA. The study was conducted at 147 sites in seven countries (USA, Canada, Germany, Italy, Japan, Spain and Vietnam) between 12 January 2022 and 26 February 2025. At Visit 1, eligible participants initiated a twice-daily BD 320 μg *via* MDI (BD 320) run-in period. At Visit 4, participants were randomised (1:2:2:2) to twice-daily BFF 160/10 μg (BFF_A_ 160), BFF_A_ 320/10 μg (BFF_A_ 320) or BD 320, *via* MDIs using Aerosphere co-suspension delivery technology, or open-label BFF 320/9 μg *via* dry-powder inhaler (BFF_DPI_; Symbicort Turbuhaler). The BFF_A_ 160 arm was included to support exploratory analyses. BFF_DPI_ was open-label and included only as a standard-of-care comparator. Randomisation was stratified by baseline asthma treatment (ICS *versus* ICS/LABA) and age (12– <18 *versus* ≥18 years). Participants had four additional in-clinic treatment visits and two telemedicine visits over the 24-week treatment period. The study ended when the last randomised participant completed 24 weeks of randomised study treatment and a 2-week safety follow-up phone call.

The study was conducted in accordance with the ethical principles of the Declaration of Helsinki and consistent with International Council for Harmonisation Good Clinical Practice (GCP), applicable regulatory requirements and the study sponsor's policies on bioethics. The study protocol and participant informed consent documents were reviewed and approved by local or country-specific controlling institutional review boards or independent ethics committees. Protocol details may be available upon request to the corresponding author. Participants or their legally authorised representatives were required to sign informed consent statements, with participants informed that their involvement was voluntary. As the study was conducted during the COVID-19 pandemic, study continuity measures were put into place to ensure pandemic-related disruptions were minimal.

### Population

Detailed inclusion and exclusion criteria are provided in supplementary table S1. In brief, participants were aged 12–80 years, with a documented history of inadequately controlled physician-diagnosed asthma for ≥6 months (ACQ-7 total score ≥1.5), pre-bronchodilator forced expiratory volume in 1 s (FEV_1_) <90% predicted normal and pre-dose FEV_1_ 50–90% predicted. Additionally, participants had to be regularly using stable daily medium-dose ICS or ICS/LABA regimen for ≥8 weeks and exhibit reversibility to salbutamol, defined as post-salbutamol increase in FEV_1_ of ≥12% and ≥200 mL in those aged ≥18 years or post-salbutamol increase in FEV_1_ of ≥12% in those 12– <18 years of age, either in the 12 months before Visit 1 or at Visit 2 or Visit 3.

### End-points

Due to regional health authority requirements, different end-points and comparators were assessed in three regional regulatory approaches for the USA, Europe/Rest of World (RoW) and Japan. Data from all participants recruited across all geographies were used in the submission approaches, which differ by comparator, end-points and multiple testing approach used according to the health authority responsible for reviewing marketing authorisation, not participants’ recruitment location or nationality. All prespecified analysis results are reported in line with the CONSORT (CONsolidated Standards Of Reporting Trials) guidelines (www.equator-network.org/reporting-guidelines/consort).

Here we report the results of the US regional approach in the main text, with findings based on the Europe/RoW and Japan regional approaches reported in the supplementary material. Primary end-points were change from baseline in FEV_1_ area under the curve from 0 to 3 h (AUC_0–3_) at week 24 for the US regional approach and change from baseline in morning pre-dose (trough) FEV_1_ over 24 weeks in the Europe/RoW regional approach or over 12–24 weeks for the Japan regional approach. Predefined subgroup analyses stratified participants by demographics (age, sex, race and region) and clinical characteristics (baseline severe asthma exacerbation history, baseline asthma treatment and percent reversibility).

Key secondary end-points included change from baseline in morning pre-dose trough FEV_1_ at week 24 (US regional approach) and change from baseline in FEV_1_ AUC_0–3_ over 24 weeks (Europe/RoW regional approach) or over 12–24 weeks (Japan regional approach). Additional secondary end-points included: onset of action (change in FEV_1_ at 5 min post-dose on day 1), responder analyses of patient-reported outcome (PRO) tools (comprising ACQ-7, ACQ-5 and Asthma Quality of Life Questionnaire for 12 years and older (AQLQ(s)+12)) at week 24 (US approach); at week 24, over 24 weeks or over 12–24 weeks (depending on the regional approach); rate of severe exacerbations (VATHOS only and VATHOS+LITHOS combined; US regional approach only); and change from baseline in the number of rescue medication inhalations over 24 weeks (12–24 weeks for Japan regional approach). Safety assessments included the occurrence of AEs and change from baseline in vital signs and clinical laboratory parameters.

In addition to the prespecified analysis of BFF_A_ *versus* BD (superiority), for the Japanese regulatory approach, non-inferiority analyses for BFF_A_ *versus* BFF_DPI_ were also conducted using data for all study patients in the Per-Protocol Analyses Set in a prespecified manner for primary and key secondary lung function end-points (using a Principal Stratum estimand in a Per-Protocol Analyses Set over 12–24 weeks). *Post hoc* analyses of BFF_A_ *versus* BFF_DPI_ were performed in the Efficacy Analysis Set using the primary estimand strategy with the same non-inferiority margins as BFF_A_ *versus* BFF_DPI_ for lung function end-points at 24 weeks (pre-dose trough FEV_1_: −100 mL; FEV_1_ AUC_0–3_: −125 mL), as well as comparisons of BFF_DPI_ *versus* BD at 24 weeks to provide additional context.

### Statistical analyses

For change from baseline in FEV_1_ AUC_0–3_, the target sample size (see figure 1 for target randomisation numbers) provided 99% power to detect a statistically significant difference at week 24 (two-sided α=0.05), assuming a true difference of 192 mL (sd 350 mL) between treatments. For change from baseline in morning pre-dose trough FEV_1_, this sample size provided 84% power to detect a statistically significant difference at week 24 (two-sided α=0.05), assuming a true difference of 112 mL (sd 320 mL) between treatments. These thresholds were set based on prior clinical studies evaluating BFF and adjusted based on predicted rates of dropout and missing data [4, 6, 13, 14]. Multiple testing procedures (MTPs) were applied to meet the regional registrational requirements (supplementary figure S1). To account for multiplicity, a combination of hierarchical testing and the Hochberg approach was followed to control the overall Type I error rate. p-values <0.05 but not statistically significant, due to either placement in or absence from the MTP, were noted as nominal. The MTP for the US regional approach was applied to both VATHOS and LITHOS to allow data from both studies to be combined for analyses of severe exacerbations. Severe exacerbations were assessed over 12 weeks in LITHOS and over 24 weeks in VATHOS.

All observed data were used unless participants received new asthma medication in conjunction with study treatment discontinuation (considered an unfavourable response). Full details of the statistical analyses for all primary and secondary end-points can be found in the supplementary material. In brief, efficacy analyses were conducted in the Efficacy Set (all randomised participants who received any treatment). Change from baseline in FEV_1_ AUC_0–3_ and in morning pre-dose trough FEV_1_ were analysed using a repeated measures ANCOVA model. The model included treatment, visit, prior maintenance medication (ICS *versus* ICS/LABA) and treatment-by-visit interaction as categorical covariates, with baseline trough FEV_1_ and percent salbutamol reversibility included as continuous covariates. Onset of action was assessed using a repeated measures ANCOVA fitted to the day 1 post-baseline data. Severe exacerbation rate (US regional approach only) was assessed using a negative binomial regression model, whereas the pooled VATHOS+LITHOS analysis used a negative binomial additive model, with study (LITHOS or VATHOS), budesonide dose (160 or 320 μg) and formoterol dose (0 or 9.6 μg) included as covariates in place of treatment. Log time at risk of experiencing a severe exacerbation was used as an offset variable in the model. For PROs, the percentage of responders was assessed using a logistic regression model, while change from baseline in rescue medication use was assessed using a repeated measures ANCOVA model. Safety analyses were conducted in the Safety Set (all randomised participants who received any treatment); data are reported descriptively, with no hypothesis testing performed. All efficacy end-points by regional approach are listed in supplementary table S2.

## Results

**FIGURE 2:**
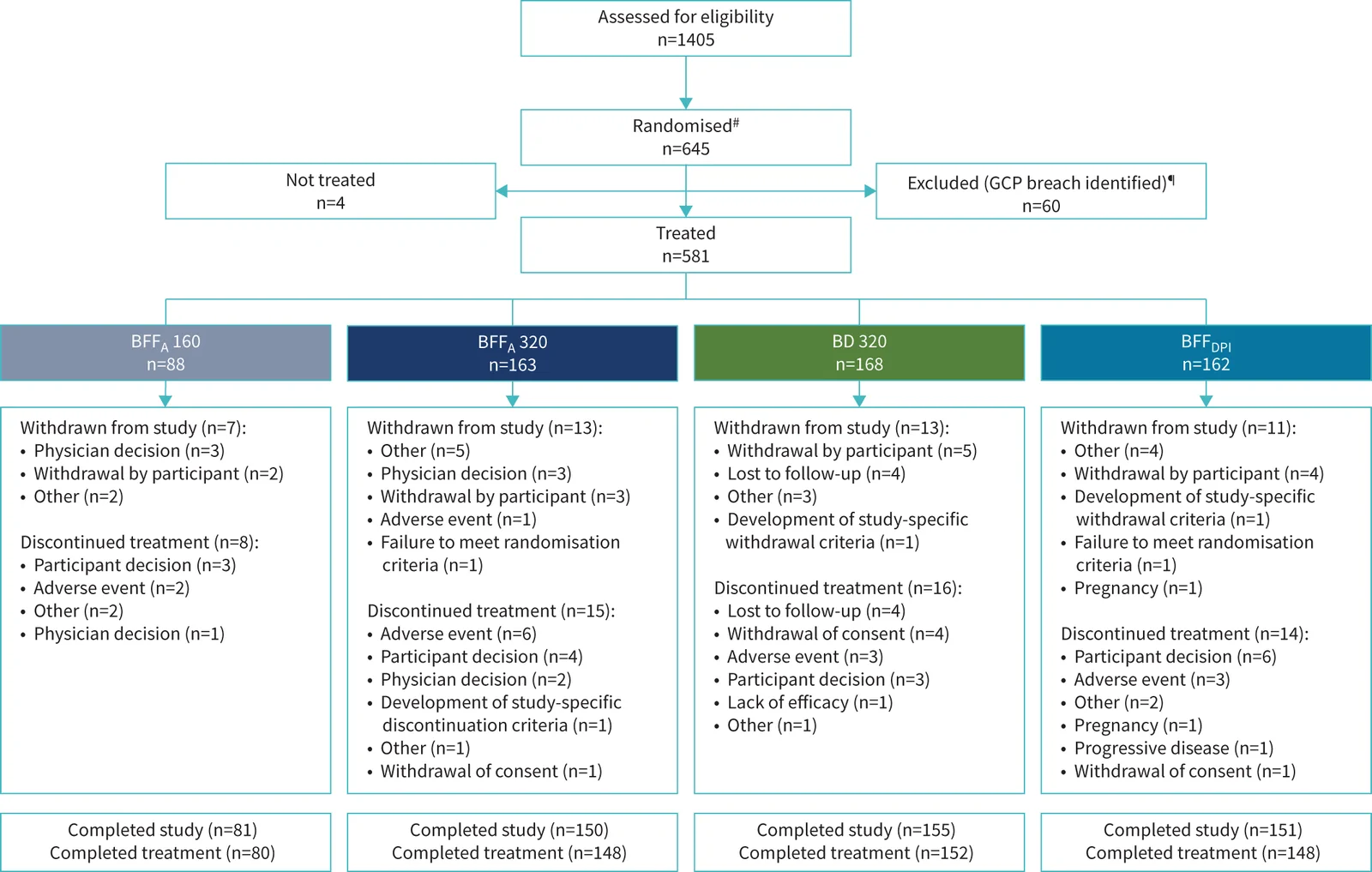
Participant disposition. ^#^: participants were randomised using a centralised Interactive Response Technology (IRT) system. The participant, care provider, investigator and outcomes assessor were blinded during the study, with the IRT programmed with blind-breaking instructions, unless safety concerns or a regulatory requirement necessitated unblinding. ^¶^: Good Clinical Practice (GCP) violations were identified in one site in the USA and in some sites in Japan. Data from affected participants were excluded from all analysis sets except the Screened Set. Reasons for withdrawal categorised as “other” included participant discontinuation after randomisation, participant discontinued study but did not withdraw consent, technical difficulties with testing, asthma exacerbation that required therapy enhancing, participant decision, participant relocation and study site closure. BD 320: budesonide 320 μg *via* Aerosphere MDI; BFF_A_ 160: budesonide/formoterol fumarate dihydrate 160/10 μg *via* Aerosphere MDI; BFF_A_ 320: budesonide/formoterol fumarate dihydrate 320/10 μg *via* Aerosphere MDI; BFF_DPI_: budesonide/formoterol fumarate dihydrate 320/9 μg *via* dry-powder inhaler; MDI: metered-dose inhaler.

Participants were enrolled in the study between 12 January 2022 and 9 August 2024, and the last participant visit was 26 February 2025. Of 645 randomised participants, 581 were treated (BFF_A_ 160, n=88; BFF_A_ 320, n=163; BD 320, n=168; BFF_DPI_, n=162) and 537 completed the study (BFF_A_ 160, n=81; BFF_A_ 320, n=150; BD 320, n=155; BFF_DPI_, n=151) (figure 2). 53 participants discontinued study medication (BFF_A_ 160, n=8; BFF_A_ 320, n=15; BD 320, n=16; BFF_DPI_, n=14), with the most common reason for discontinuation being participant decision (n=16). During the study, serious GCP violations were discovered at several sites in Japan and one site in the USA; affected participants were withdrawn from the study or considered screen failures, the affected sites were terminated and data from participants at affected sites were excluded from all analysis sets except the Screened Set (all participants who provided consent to participate).

Baseline demographics were generally well balanced (table 1). Most participants were female (55.2–65.1%) and aged 18–65 years (75.9–78.3%). In terms of baseline clinical characteristics, mean reversibility ranged from 17.5% to 20.0%, mean percent predicted pre-bronchodilator FEV_1_ ranged from 70.4% to 72.2% predicted and the majority of participants had received prior ICS/LABA, ranging from 87.3% to 89.5%, across treatment groups.

**TABLE 1:** Demographics and clinical characteristics: Efficacy Set

	BFF_A_ 160 (n=87)	BFF_A_ 320 (n=162)	BD 320 (n=166)	BFF_DPI_ (n=160)
**Age (years)**	49.4±17.2	49.7±15.8	49.0±15.2	51.3±15.3
**Age group**				
≥12 to <18 years	3 (3.4)	6 (3.7)	7 (4.2)	5 (3.1)
≥18 to <65 years	67 (77.0)	123 (75.9)	130 (78.3)	122 (76.3)
≥65 to <75 years	14 (16.1)	30 (18.5)	28 (16.9)	30 (18.8)
≥75 years	3 (3.4)	3 (1.9)	1 (0.6)	3 (1.9)
**Sex**				
Male	39 (44.8)	61 (37.7)	58 (34.9)	59 (36.9)
Female	48 (55.2)	101 (62.3)	108 (65.1)	101 (63.1)
**Race**				
White	64 (73.6)	119 (73.5)	122 (73.5)	112 (70.0)
Asian	9 (10.3)	22 (13.6)	18 (10.8)	19 (11.9)
Black or African American	9 (10.3)	16 (9.9)	17 (10.2)	24 (15.0)
American Indian or Alaskan Native	0 (0.0)	0 (0.0)	1 (0.6)	0 (0.0)
Native Hawaiian or Other Pacific Islander	0 (0.0)	0 (0.0)	0 (0.0)	1 (0.6)
Other	5 (5.7)	5 (3.1)	8 (4.8)	4 (2.5)
**Baseline percent reversibility (%)^#^**	18.5±11.3	17.5±12.8	19.9±11.0	20.0±15.9
**Baseline blood eosinophil count (cells·mm^−3^)**	196.8±132.6	201.7±276.3	216.5±255.6	200.7±155.2
**Baseline blood eosinophil count**				
<150 cells·mm^−3^	39 (44.8)	76 (46.9)	79 (47.6)	79 (49.4)
≥150 to <300 cells·mm^−3^	32 (36.8)	55 (34.0)	52 (31.3)	48 (30.0)
≥300 cells·mm^−3^	16 (18.4)	28 (17.3)	35 (21.1)	33 (20.6)
Missing	0 (0.0)	3 (1.9)	0 (0.0)	0 (0.0)
**Baseline pre-bronchodilator FEV_1_ (% pred)**	72.2±9.8	70.7±9.7	71.3±9.6	70.4±9.8
**Baseline pre-bronchodilator FEV_1_ (mL)**	2315±666	2163±666	2132±561	2103±589
**Prior asthma medication use**				
ICS	11 (12.6)	17 (10.5)	20 (12.0)	19 (11.9)
ICS/LABA	76 (87.4)	145 (89.5)	145 (87.3)	141 (88.1)
ICS/LAMA/LABA	0 (0.0)	0 (0.0)	1 (0.6)	0 (0.0)
**Baseline ACQ-7 score^¶^**	2.2±0.5	2.3±0.5	2.4±0.6	2.3±0.5
**Severe exacerbations within prior year (n)**	0.2±0.4	0.2±0.5	0.2±0.4	0.2±0.4
**Severe exacerbation events in 12 months before Visit 1**				
0	73 (83.9)	134 (82.7)	139 (83.7)	137 (85.6)
≥1	14 (16.1)	28 (17.3)	27 (16.3)	23 (14.4)

Data are presented as mean±sd or n (%). ACQ: Asthma Control Questionnaire; BD 320: budesonide 320 μg *via* Aerosphere MDI; BFF_A_ 160: budesonide/formoterol fumarate dihydrate 160/10 μg *via* Aerosphere MDI; BFF_A_ 320: budesonide/formoterol fumarate dihydrate 320/10 μg *via* Aerosphere MDI; BFF_DPI_: budesonide/formoterol fumarate dihydrate 320/9 μg *via* dry-powder inhaler; FEV_1_: forced expiratory volume in 1 s; ICS: inhaled corticosteroid; LABA: long-acting β_2_-agonist; LAMA: long-acting muscarinic antagonist; MDI: metered-dose inhaler. ^#^: reversibility (%) was calculated as (post-salbutamol FEV_1_−pre-salbutamol FEV_1_)/pre-salbutamol FEV_1_×100. Visit 2 values were used if non-missing, otherwise Visit 3 values were used. Reversibility at screening included only participants who were randomised based on reversibility at Visit 2 or Visit 3. ^¶^: baseline ACQ-7 was defined as the last non-missing ACQ value before randomisation.

### Primary and key secondary end-points

**FIGURE 3:**
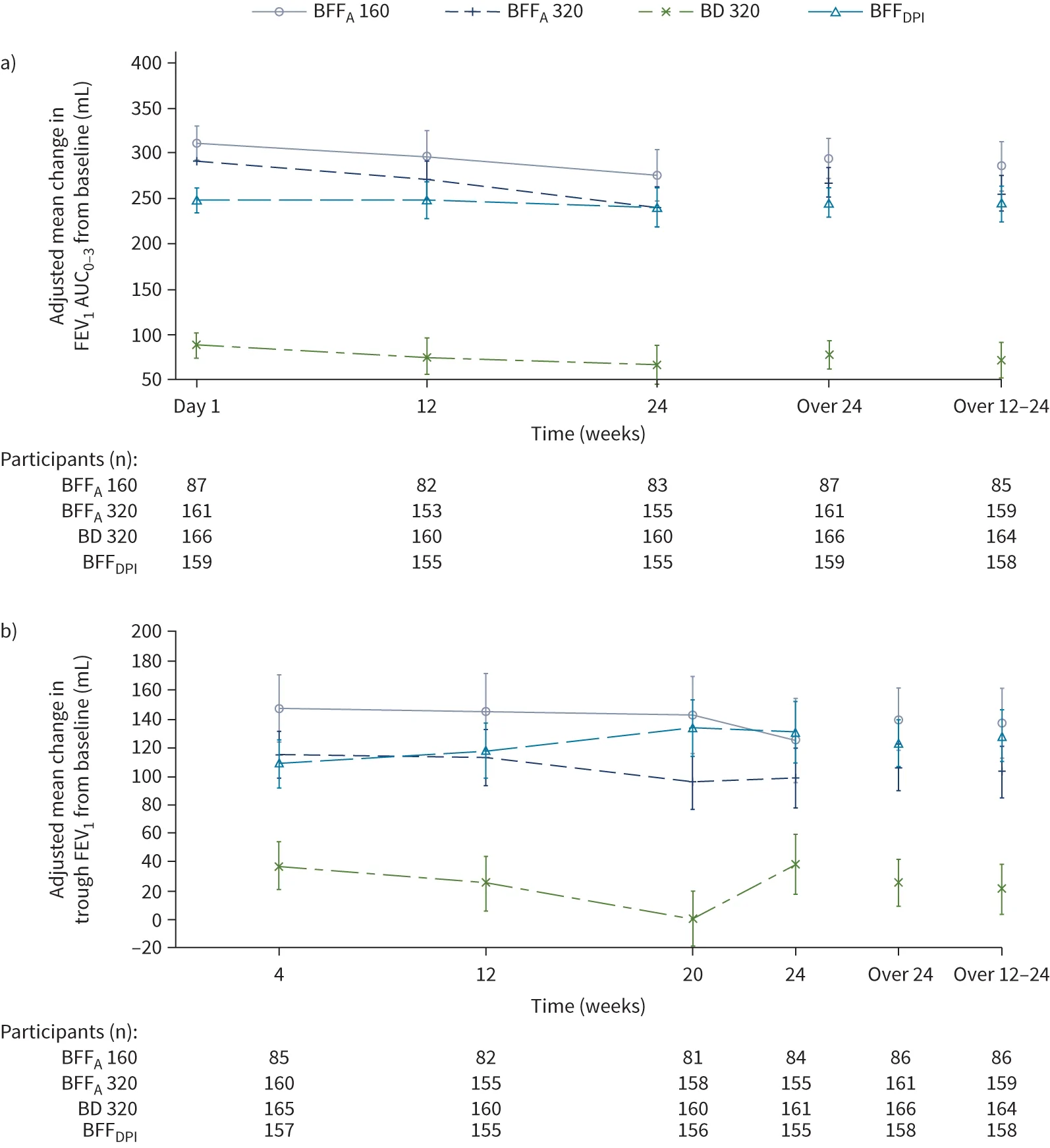
Change from baseline over time for a) forced expiratory volume in 1 s area under the curve from 0 to 3 h (FEV_1_ AUC_0–3_) and b) morning pre-dose trough FEV_1_ (Efficacy Set). Analysed using a repeated measures ANCOVA model. For further details on models and covariates used, see the supplementary material. BD 320: budesonide 320 μg *via* Aerosphere MDI; BFF_A_ 160: budesonide/formoterol fumarate dihydrate 160/10 μg *via* Aerosphere MDI; BFF_A_ 320: budesonide/formoterol fumarate dihydrate 320/10 μg *via* Aerosphere MDI; BFF_DPI_: budesonide/formoterol fumarate dihydrate 320/9 μg *via* dry-powder inhaler; MDI: metered-dose inhaler.

At week 24, BFF_A_ 320 demonstrated a statistically significant greater improvement in mean change from baseline in FEV_1_ AUC_0–3_ (least squares (LS) mean difference 173 (95% CI 112–233) mL; p<0.0001) and trough FEV_1_ (61 (95% CI 1–120) mL; p=0.0447) *versus* BD 320 (table 2 and figure 3). Comparable findings were observed over 24 weeks and 12–24 weeks in the Europe/RoW and Japan regional approaches, respectively (supplementary table S3), and across subgroups (supplementary figure S2).

**TABLE 2:** Summary of efficacy end-points: Efficacy Set^#^ (US regional approach)

	BFF_A_ 320	BD 320	BFF_DPI_
**Primary end-point**			
Change from baseline in FEV_1_ AUC_0–3_ at week 24^¶^ (mL)	n=155	n=160	n=155
Estimate (se)	240 (21.9)	67 (21.6)	240 (22.0)
LS mean difference (95% CI); p-value (comparison *versus* BD)	**173 (112 to 233); p<0.0001** ^+^	Comparator	173 (113 to 234); p<0.0001
LS mean difference (95% CI); p-value (comparison BFF_A_ *versus* BFF_DPI_)	0 (−61 to 61); p=0.9903	NA	Comparator
**Secondary end-points included in the Type I error control procedure**			
Key secondary end-point: change from baseline in trough FEV_1_ at week 24^¶^ (mL)	n=155	n=161	n=155
Estimate (se)	99 (21.5)	38 (21.1)	131 (21.6)
LS mean difference (95% CI); p-value (comparison *versus* BD)	**61 (1 to 120); p=0.0447** ^+^	Comparator	93 (33 to 152); p=0.0023
LS mean difference (95% CI); p-value (comparison BFF_A_ *versus* BFF_DPI_)	−32 (−92 to 28); p=0.2962	NA	Comparator
Onset of action: change in FEV_1_ at 5 min post-dose on day 1^§^ (mL)	n=159	n=158	n=156
Estimate (se)	184 (12.2)	41 (12.1)	161 (12.3)
LS mean difference (95% CI); p-value (comparison *versus* BD)	**144 (110 to 177); p<0.0001** ^+^	Comparator	120 (86 to 154); p<0.0001
LS mean difference (95% CI); p-value (comparison BFF_A_ *versus* BFF_DPI_)	−24 (−58 to 10); p=0.1727	NA	Comparator

Bold text indicates prespecified analyses; non-bold text indicates *post hoc* analyses.

ACQ: Asthma Control Questionnaire; AQLQ(s)+12: Asthma Quality of Life Questionnaire for 12 years and older; AUC_0–3_: area under the curve over 0–3 h; BD 320: budesonide 320 μg *via* Aerosphere MDI; BFF_A_ 320: budesonide/formoterol fumarate dihydrate 320/10 μg *via* Aerosphere MDI; FEV_1_: forced expiratory volume in 1 s; ICS: inhaled corticosteroid; LABA: long-acting β_2_-agonist; LS: least squares; MDI: metered-dose inhaler; NA: not applicable. ^#^: all observed data were used unless participants discontinued prematurely in conjunction with asthma therapy or prohibited medication potentially impacting efficacy, in which case treatment failure was imputed. ^¶^: analysed using a repeated measures ANCOVA model; the model included treatment, visit, prior maintenance medication (ICS *versus* ICS/LABA) and treatment-by-visit interaction as categorical covariates, and baseline trough FEV_1_ and percent reversibility as continuous covariates. For further details on models and covariates used, see the supplementary material. ^+^: significant per the Type I error control procedure. ^§^: assessed using a repeated measures ANCOVA fitted to day 1 post-baseline data.

Non-inferiority of BFF_A_ *versus* BFF_DPI_ was concluded based on prespecified analyses of data from all participants according to the Japanese regulatory approach (over 12–24 weeks, using a Principal Stratum estimand in a Per-Protocol Analysis Set) (supplementary table S2). *Post hoc* comparisons of BFF_DPI_ *versus* BD and BFF_A_ *versus* BFF_DPI_ in the Efficacy Analysis Set at week 24 are summarised in table 2. BFF_DPI_, included as an active comparator, demonstrated improvements *versus* BD. Non-inferiority was also observed by applying the same margins to *post hoc* comparisons of BFF_A_ *versus* BFF_DPI_ in the Efficacy Analysis Set using the primary estimand strategy. Descriptive statistics of lung function end-points are shown in supplementary table S4.

### Secondary end-points

Onset of action was significantly greater with BFF_A_ 320 *versus* BD 320 (LS mean difference 144 (95% CI 110–177) mL; p<0.0001) (table 2 and supplementary figure S3). The proportion of ACQ-7 and ACQ-5 responders (≥0.5 total score decrease from baseline) at week 24 was not significantly different between BFF_A_ 320 and BD 320 (table 3), with similar findings for Europe/RoW and Japan (supplementary table S3). AQLQ(s)+12 results were nominal due to placement in MTPs after hierarchical failure of a preceding end-point (supplementary figure S1). The supportive tertiary end-point of change from baseline in the ACQ-7 score was nominally improved with BFF_A_ 320 *versus* BD 320 (LS mean difference −0.191 (95% CI –0.360 to −0.022)). For AQLQ(s)+12, the percentage of responders (≥0.5 total score increase from baseline) was nominally improved with BFF_A_ 320 *versus* BD 320 at week 24 (p=0.019) (table 3), and over 24 weeks in the Europe/RoW analysis (p=0.0233), but not over 12–24 weeks in the analysis for Japan (p=0.0861) (supplementary table S3). The change from baseline in ACQ-5 and AQLQ(s)+12 for BFF_A_ 160, BFF_A_ 320 and BFF_DPI_ showed greater improvement compared with BD 160 as early as 4 weeks, and improvement remained throughout the 24-week time course (supplementary figure S5).

**TABLE 3:** Summary of secondary end-points: Efficacy Set^#^ (US regional approach)

	BFF_A_ 320	BD 320
**Percentage of ACQ-7 responders at week 24^¶^**	n=155	n=161
Responders (n (%))	106 (68.4)	101 (62.7)
Odds ratio (95% CI); p-value	1.283 (0.803 to 2.052); p=0.2977	
**Percentage of ACQ-5 responders at week 24^¶^**	n=155	n=161
Responders (n (%))	111 (71.6)	106 (65.8)
Odds ratio (95% CI); p-value	1.332 (0.819 to 2.166); p=0.2477	
**Percentage of AQLQ(s)+12 responders at week 24^¶^**	n=149	n=157
Responders (n (%))	86 (57.7)	74 (47.1)
Odds ratio (95% CI); p-value	1.770 (1.097 to 2.858); p=0.0194^+^	
**Change from baseline in mean rescue medication use over 24 weeks^§^ (inhalations per day)**	n=157	n=163
Estimate (se)	−0.481 (0.0781)	−0.213 (0.0764)
Mean difference (95% CI); p-value	−0.269 (−0.482 to −0.053); p=0.146	

ACQ: Asthma Control Questionnaire; AQLQ(s)+12: Asthma Quality of Life Questionnaire for 12 years and older; BD 320: budesonide 320 μg *via* Aerosphere MDI; BFF_A_ 320: budesonide/formoterol fumarate dihydrate 320/10 μg *via* Aerosphere MDI. ^#^: all observed data were used unless participants discontinued prematurely in conjunction with asthma therapy or prohibited medication potentially impacting efficacy, in which case treatment failure was imputed. ^¶^: assessed using logistic regression. ^+^: nominally significant but not statistically significant due to the Type I error control procedure. ^§^: assessed using a repeated measures ANCOVA model. The model included treatment, the number of the relevant 4-week interval (interval 1 to 6), prior maintenance medication (ICS *versus* ICS/LABA), severe asthma exacerbation history (0, ≥1) and the treatment-by-4-week interval interaction as categorical covariates, and baseline daily rescue salbutamol use, baseline trough FEV_1_ and percent salbutamol reversibility as continuous covariates.

With regard to rescue medication use, BFF_A_ 320 provided a nominally greater reduction from baseline in mean daily inhalations *versus* BD 320 when assessed over 24 weeks for both the US and Europe/RoW regional approaches (p=0.0146) (table 3). The results were similar for the Japan regional approach, assessed over 12–24 weeks (supplementary table S3). The study was not designed for dose response evaluation; however, both doses demonstrated similar improvements on lung function from baseline (supplementary table S5). The tertiary/exploratory end-point of percentage of symptom-free days over 24 weeks while on treatment was higher with BFF_A_ 320 *versus* BD 320 and was nominally significant (supplementary table S6).

When comparing severe exacerbation rates with BFF_A_ 320 *versus* BD 320, the rate ratios were not statistically significant for either VATHOS alone (rate ratio 1.02 (95% CI 0.61–1.70); p=0.9473) or pooled VATHOS+LITHOS (rate ratio 0.82 (95% CI 0.54–1.25); p=0.3610) (table 4). Since asthma is an inflammatory disease, *post hoc* generalised additive model plots were generated for the change in baseline over efficacy end-points change from baseline in FEV_1_ AUC_0–3_ and change from baseline in morning pre-dose trough FEV_1_ at week 24 *versus* baseline eosinophil count, for BFF_A_ 320 and BD 160 (supplementary figure S4). While confidence intervals are wide due to limited sample sizes at different baseline eosinophil counts, the results suggest improvements in lung function with BFF_A_ *versus* BD occur generally across the range of eosinophil blood counts.

**TABLE 4:** Rate of severe exacerbations: Efficacy Set^#^

	VATHOS only		Pooled VATHOS+LITHOS	
	BFF_A_ 320 (n=161)	BD 320 (n=166)	BFF_A_ 320+BFF_A_ 160 (n=425)	BD 320+BD 160 (n=335)
**Participants experiencing exacerbations (n (%))**	25 (15.4)	27 (16.3)	48 (11.3)	48 (14.3)
**Number of exacerbations (n)**	30	29	53	51
**Total follow-up time (years)**	72.7	75.6	153.4	114.1
**Adjusted annual exacerbation rate (se)**	0.36 (0.07)	0.35 (0.07)	0.35 (0.05)	0.42 (0.07)
**Rate ratio (95% CI); p-value**	1.02 (0.61 to 1.70); p=0.9473^¶^		0.82 (0.54 to 1.25); p=0.3610^+^	

BD 160: budesonide 160 μg *via* Aerosphere MDI; BD 320: budesonide 320 μg *via* Aerosphere MDI; BFF_A_ 160: budesonide/formoterol fumarate dihydrate 160/10 μg *via* Aerosphere MDI; BFF_A_ 320: budesonide/formoterol fumarate dihydrate 320/10 μg *via* Aerosphere MDI; FEV_1_: forced expiratory volume in 1 s; FF: formoterol fumarate; ICS: inhaled corticosteroid; LABA: long-acting β_2_-agonist; MDI: metered-dose inhaler. ^#^: all observed data were used unless participants discontinued prematurely in conjunction with asthma therapy or prohibited medication potentially impacting efficacy, in which case treatment failure was imputed. ^¶^: used a negative binomial regression model, including treatment, baseline trough FEV_1_, percent salbutamol reversibility, baseline severe asthma exacerbation history (0, ≥1), prior maintenance medication (ICS *versus* ICS/LABA) and region as covariates. The time at risk of experiencing a severe exacerbation was used as an offset variable. ^+^: used a negative binomial additive model, with the same covariates as the VATHOS study-only model but additionally including study and BD dose (1 if the BD dose is 320 μg and 0 if the BD dose is 160 μg) and FF dose (1 if the FF dose is 9.6 μg and 0 if no FF dose) instead of treatment as covariates. Log time at risk of experiencing a severe exacerbation was used as an offset variable.

### Safety

No substantive differences were observed in the occurrence of on-treatment AEs, serious adverse events (SAEs) or AEs leading to discontinuation across treatments, and the most frequently reported AE across treatments was nasopharyngitis (table 5 and supplementary table S7). No on-treatment SAEs, including those with the outcome of death, were reported in either treatment group (table 5). Notably, no paradoxical bronchospasms were reported during the study. Furthermore, no clinically meaningful trends in clinical laboratory parameters, vital signs and physical findings were observed with BFF_A_ 320 treatment and values were similar to BD 320.

**TABLE 5:** Summary of overall adverse events (AEs) and most frequently reported AEs: Safety Set

	BFF_A_ 160 (n=88)	BFF_A_ 320 (n=163)	BD 320 (n=168)	BFF_DPI_ (n=162)
**Overall AEs**				
Any AE	44 (50.0)	82 (50.3)	64 (38.1)	74 (45.7)
Any SAE	3 (3.4)	3 (1.8)	1 (0.6)	7 (4.3)
Any SAE with outcome of death	0 (0)	0 (0)	0 (0)	0 (0)
Any AE leading to discontinuation of study drug	1 (1.1)	6 (3.7)	3 (1.8)	3 (1.9)
**Most frequently reported AEs^#^**				
Nasopharyngitis	12 (13.6)	15 (9.2)	15 (8.9)	13 (8.0)
COVID-19	3 (3.4)	15 (9.2)	8 (4.8)	11 (6.8)
Asthma	2 (2.3)	4 (2.5)	1 (0.6)	1 (0.6)
Bronchitis bacterial	2 (2.3)	2 (1.2)	2 (1.2)	2 (1.2)
Hypertension	3 (3.4)	1 (0.6)	3 (1.8)	1 (0.6)
Arthralgia	3 (3.4)	1 (0.6)	1 (0.6)	2 (1.2)
Rhinitis allergic	1 (1.1)	4 (2.5)	0 (0)	1 (0.6)
Diarrhoea	2 (2.3)	0 (0)	1 (0.6)	0 (0)
Gout	2 (2.3)	1 (0.6)	0 (0)	0 (0)
Influenza-like illness	2 (2.3)	0 (0)	0 (0)	0 (0)

Data are presented as n (%). BD 320: budesonide 320 μg *via* Aerosphere MDI; BFF_A_ 160: budesonide/formoterol fumarate dihydrate 160/10 μg *via* Aerosphere MDI; BFF_A_ 320: budesonide/formoterol fumarate dihydrate 320/10 μg *via* Aerosphere MDI; BFF_DPI_: budesonide/formoterol fumarate dihydrate 320/9 μg *via* dry-powder inhaler; MDI: metered-dose inhaler; SAE: serious adverse event. ^#^: occurring in ≥2% of participants in any treatment group and more common in participants treated with either BFF MDI *versus* any comparator; by Preferred Term (MedDRA (Medical Dictionary for Regulatory Activities) Version 27.1); participants with multiple occurrences in the same category were counted once per category/Preferred Term regardless of the number of occurrences.

## Discussion

In this 24-week phase 3 study assessing the efficacy and safety of medium-dose BFF delivered *via* a novel co-suspension technology in adolescents and adults, BFF_A_ 320 was found to be superior to BD 320 across primary and key secondary lung function end-points, and across regional approaches for the USA, Europe/RoW and Japan. As might be expected based on each therapy's drug composition, onset of action (day 1 change in FEV_1_, 5 min post-dose) was significantly greater with BFF_A_ 320 *versus* BD 320. There were no meaningful differences in improvement across the prespecified efficacy subgroups, stratified by demographics (such as age groups) and clinical characteristics (such as percent reversibility), where there were sufficient participants for analysis. Nominal improvements were also observed on health-related quality of life (AQLQ(s)+12) and rescue medication use with BFF_A_ 320 *versus* BD 320. The severe exacerbation rate was similar between BFF_A_ 320 and BD 320 in both VATHOS and pooled VATHOS+LITHOS, and there were no new or unexpected tolerability or safety findings with BFF_A_ 320 among the participants included in this study. Finally, no clinically meaningful trends were observed for clinical laboratory data, vital signs or physical findings.

The lung function outcomes in VATHOS are consistent with previous studies that have demonstrated greater improvements with dual ICS/LABA *versus* ICS alone in individuals diagnosed with asthma [1, 5]. Notably, the BFF_A_ 320 arm in VATHOS used the same budesonide molecule and dose and a very similar formoterol dose as that used in the currently approved BFF_DPI_ (Symbicort) Turbuhaler [15]. Therefore, the key difference is the use of the Aerosphere co-suspension delivery technology that has already been demonstrated to provide improved drug delivery in people with COPD [9]. As minimal clinically important differences in asthma, including the meaningful change in post-dose FEV_1_, are contentious based on a lack of clarity around anchors and population (especially now that ICS is benchmarked as the initiating therapy and subsequent bronchodilators are added to those already established on ICS [16–19]), they were not investigated in this study.

Data from real-world studies demonstrate that the high symptom burden associated with inadequately controlled asthma negatively impacts patient health status, productivity and absenteeism [20, 21]. While improvements in AQLQ(s)+12 and rescue medication in VATHOS were nominal only, they nonetheless indicate that participants receiving BFF_A_ 320 experienced some benefits in symptom burden and quality of life compared with those receiving BD 320.

The rate of severe exacerbations with BFF_A_ 320 was not significantly different from the rate for BD 320 in either VATHOS alone or in the prespecified pooled VATHOS+LITHOS data. However, as the study populations were not selected based on exacerbation risk, neither VATHOS nor LITHOS were powered for this analysis, and it is therefore not surprising that the overall numbers of participants experiencing exacerbations during the studies were low. In addition, the pooling of studies to evaluate severe exacerbations had to account for the different doses of budesonide used in VATHOS and LITHOS, although there is precedent for this type of pooled analysis in the TRIMARAN and TRIGGER studies [22].

Other limitations include the relatively short duration of VATHOS (24 weeks), particularly with respect to exacerbation risk: a longer treatment duration (up to 52 weeks) may be better suited to assess BFF treatment effects. Additionally, to ensure clinical equipoise for participants who entered while receiving ICS/LABA and were subsequently randomised to an ICS alone, and to optimise for the evaluation of the key lung function end-points, participants were required to have demonstrated asthma stability based on symptom worsening assessments during screening. This is a limitation for evaluating severe exacerbations, as excluding asthma instability and not requiring exacerbations in the prior year limits the number of exacerbation events expected and subsequent ability to demonstrate a treatment effect on asthma exacerbations.

Overall, these findings support the use of BFF_A_ 320 delivered *via* a novel co-suspension delivery technology (Aerosphere) for the treatment of people with asthma who remain inadequately controlled despite treatment with medium-dose ICS or ICS/LABA.

## Data Availability

Data underlying the findings described in this article may be obtained in accordance with AstraZeneca's data sharing policy described at https://astrazenecagrouptrials.pharmacm.com/ST/Submission/Disclosure. Data for studies directly listed on Vivli can be requested through Vivli at www.vivli.org. Data for studies not listed on Vivli could be requested through Vivli at https://vivli.org/members/enquiries-about-studies-not-listed-on-the-vivli-platform. The AstraZeneca Vivli member page is also available outlining further details: https://vivli.org/ourmember/astrazeneca.

## Supplementary material

**Please note:** supplementary material is not edited by the Editorial Office, and is uploaded as it has been supplied by the author. Supplementary material 01817-2025.SUPPLEMENT
