# Supplementary material for: Budesonide/formoterol fumarate dihydrate versus budesonide with a novel suspension technology for uncontrolled asthma: phase 3 VATHOS study
Source: ERJ Open Res. 2026 Sep 14;12(5):01817-2025. doi: 10.1183/23120541.01817-2025 (PMC13573227; doi:10.1183/23120541.01817-2025)
Supplement: Supplementary file 1 [file 01817-2025.SUPPLEMENT.pdf]

SUPPLEMENTARY INFORMATION

Budesonide/formoterol fumarate dihydrate vs budesonide with a novel suspension technology for uncontrolled asthma: Phase 3 VATHOS study

David J. Jackson[1], Robert A. Wise[2], Alberto Papi[3], Njira Lugogo[4], Ruchong Chen[5], Hassan Chami[2,6], Clara Armengol[7], Sami Z. Daoud[6], Emma Nauclér[8], Karin Bowen[9], Ayman Megally[6], Olami Sobande[6] and Mehul Patel[10]

[1]Guy's Severe Asthma Centre, Guy's Hospital, School of Immunology & Microbial Sciences, King's College London, London, UK; [2]Department of Medicine, Johns Hopkins University School of Medicine, Baltimore, MD, USA; [3]Respiratory Medicine, Department of Translational Medicine, University of Ferrara, Ferrara, Italy; [4]Division of Pulmonary & Critical Care Medicine, University of Michigan, Ann Arbor, MI, USA; [5]State Key Laboratory of Respiratory Disease, Joint International Research Laboratory of Respiratory Health, National Clinical Research Center for Respiratory Disease, National Center for Respiratory Medicine, Department of Allergy and Clinical Immunology, Guangzhou Institute of Respiratory Health, The First Affiliated Hospital of Guangzhou Medical University, Guangzhou, Guangdong, P.R. China and Guangzhou National Laboratory, Guangzhou, P.R. China; [6]Respiratory and Immunology, Clinical Development, Biopharmaceuticals R&D, AstraZeneca, Gaithersburg, MD, USA; [7]Respiratory and Immunology, Clinical Development, Biopharmaceuticals R&D, AstraZeneca, Barcelona, Spain; [8]Respiratory and Immunology, Biometrics, Biopharmaceuticals R&D, AstraZeneca, Gothenburg, Sweden; [9]Respiratory and Immunology, Biometrics, Biopharmaceuticals R&D, AstraZeneca, Gaithersburg, MD, USA; [10]Respiratory and Immunology, Clinical Development, Biopharmaceuticals R&D, AstraZeneca, Cambridge, UK.

CONTENTS

Protocol amendments 3

Statistical analysis 4

Sample size (EU/ROW approach) 4

Primary endpoints 4

Secondary endpoints 4

SUPPLEMENTARY TABLE S1. Key inclusion and exclusion criteria 6

SUPPLEMENTARY TABLE S2. Efficacy endpoints in the VATHOS study by regional approach (US, EU/ROW, and Japan submission approaches; Efficacy Set*) 7

SUPPLEMENTARY TABLE S3. Summary of efficacy endpoints, (Europe/ROW regional approach and Japan regional approach), Efficacy Set (used for superiority comparisons) 9

SUPPLEMENTARY TABLE S4. Descriptive statistics of lung function endpoints, Efficacy Set[a] 13

SUPPLEMENTARY TABLE S5. Effect of BFF doses (US regional approach), Efficacy Set 15

SUPPLEMENTARY TABLE S6. Percentage of Symptom-free Days Over 24 Weeks: While on Treatment Estimand (Efficacy Set) 16

SUPPLEMENTARY TABLE S7. Summary of SAEs, Safety Set 17

SUPPLEMENTARY FIGURE S1. Overview of Type I error Control MTPs for A) US regional approach; B) Europe/ROW regional approach; and C) Japan regional approach 19

SUPPLEMENTARY FIGURE S2. Subgroup analyses of primary endpoints by regional approach: A) US (FEV_1 AUC_{0-3} at Week 24); B) Europe/ROW (trough FEV_1 over 24 weeks); and C) Japan (trough FEV_1 over 12–24 weeks.) 21

SUPPLEMENTARY FIGURE S3. Change from baseline in onset of action at 5 minutes post-dose 24

SUPPLEMENTARY FIGURE S4. Generalized additive-model plot for A) change from baseline in FEV_1 AUC_{0-3} and B) change from baseline in morning pre-dose trough FEV_1 at Week 24 25

SUPPLEMENTARY FIGURE S5. Change from baseline by timepoint in A) ACQ-5 and B) AQLQ(s)+12. 27

Protocol amendments

Amendments to the protocol were made after initiating participant recruitment. Severe asthma exacerbation rate analyses (pooled and individual studies) were added after feedback from a health authority. The Type I error control procedure for the Europe/rest of world (ROW) regional approach was updated so that the onset of action endpoint was tested separately and outside of the Hochberg test procedure. The onset of action assessment was changed to the difference in mean change from baseline, no longer performed as a within-group test, and compared BFF_A 320 with BD 320 in the multiple testing procedure (MTP); the within-group test was retained as a supportive/exploratory analysis. The Type I error control procedure for the US regional approach was updated to change the primary estimand to the treatment policy estimand, and a subsequent amendment further updated the strategies for handling intercurrent events. Strategies for handling intercurrent events were updated to reflect a composite strategy (treatment failure) if new asthma therapy or administration of prohibited medication thought to impact efficacy occurred in conjunction with premature investigational product discontinuation. Otherwise, the Treatment Policy strategy was used.

Statistical analysis

Sample size (EU/ROW approach)

A sample size of 630 participants (BFF_A 160: 90 participants; BFF_A 320, BD 320 and BFF_{DPI}: 180 participants each) was planned to assess the primary endpoints. This assumed approximately 15% of randomised participants would discontinue before Week 12, and 20%

by Week 24. For change from baseline in FEV_1 AUC_{0-3}, this sample size provided 99% power to detect a statistically significant difference over 24 weeks, assuming a true difference of 200 mL (standard deviation [SD] 308 mL) between treatments. For change from baseline in morning pre-dose trough FEV_1, this sample size provided >90% power over 24 weeks, assuming a true difference of 100 mL (SD 268 mL) between treatments.

Primary endpoints

Change from baseline in FEV_1 AUC_{0-3} was analysed using a repeated measure of covariance (ANCOVA). The model included treatment, visit, prior maintenance medication (ICS versus ICS/LABA), and treatment-by-visit interaction as categorical covariates and baseline trough FEV_1 and percent salbutamol reversibility as continuous covariates.

Secondary endpoints

The onset of action was analysed using a repeated measures ANCOVA fitted to the Day 1 post-baseline data. The model included treatment, timepoint, prior maintenance medication (ICS *versus* ICS/LABA), and treatment-by-timepoint interaction as categorical covariates and baseline trough FEV_1 and percent reversibility as continuous covariates. For the rate of severe exacerbation analysis in the US regional approach, a negative binomial regression model was used for VATHOS, with treatment, baseline trough FEV_1, percent salbutamol reversibility, baseline severe asthma exacerbation history (0, ≥1), prior maintenance medication (ICS versus ICS/LABA), and region as covariates. For the pooled VATHOS + LITHOS analysis, exacerbation rates were assessed using a negative binomial additive model with the same covariates as the VATHOS study-only model but additionally including study and BD dose (1 if the BD dose is 320 μg, and 0 if the BD dose is 160 μg) and formoterol fumarate dihydrate (FF) dose (1 if the FF dose is 9.6 μg, and 0 if no FF dose) instead of treatment as

covariates. Log time at risk of experiencing a severe exacerbation was used as an offset variable in both models.

Percentage of responders on the PROs was assessed using logistic regression to compare the treatment groups with treatment and prior maintenance medication (ICS versus ICS/LABA) as categorical covariates, and baseline instrument score, baseline trough FEV_1, and percent salbutamol reversibility as continuous covariates. Change from baseline in rescue medication use was assessed using a repeated measures ANCOVA model. The model included treatment, the number of the relevant 4-week interval (interval 1 to 6), prior maintenance medication (ICS *versus* ICS-LABA), severe asthma exacerbation history (0, ≥1) and the treatment-by-4-week interval interaction as categorical covariates, and baseline daily rescue salbutamol use, baseline trough FEV_1, and percent salbutamol reversibility as continuous covariates.

SUPPLEMENTARY TABLE S1. Key inclusion and exclusion criteria

Key inclusion criteria

- Aged 12–80 years (inclusive).
- A documented history of physician-diagnosed asthma ≥6 months before Visit 1.
- Regularly using stable daily medium-dose ICS or an ICS/LABA regimen for at least 8 weeks before Visit 1.
- A 7-item ACQ total score ≥1.5 at Visits 1 and 4.
- A pre-bronchodilator FEV_1 <90% of predicted normal at Visits 1, 2 and 3, and a pre-dose FEV_1 of 50–90% at Visit 4 (pre-randomisation).
- Exhibiting reversibility to salbutamol, defined as a post-salbutamol increase in FEV_1 of ≥12% and ≥200 mL in those ≥18 years or a post-salbutamol increase in FEV_1 of ≥12% in those 12 to <18 years of age, either in the 12 months before Visit 1 or at Visit 2 or Visit 3.
- Asthma stability during run-in based on symptom worsening assessments, including any of the following on ≥2 consecutive days: decreased morning peak expiratory flow of ≥20%, increased rescue salbutamol use, increased nighttime awakenings, or increased asthma symptoms.

Key exclusion criteria

- Life-threatening asthma, defined as a history of significant asthma episode(s) requiring intubation associated with hypercapnia, respiratory arrest, hypoxic seizures, or asthma-related syncopal episode(s).
- Hospitalisation for asthma within 8 weeks of Visit 1.
- Completed treatment for any respiratory infection or asthma exacerbation treated with systemic corticosteroids and/or additional ICS treatment in the 8 weeks prior to Visit 1 and throughout screening.
- Historical or current evidence of clinically significant disease including, but not limited to, cardiovascular, hepatic, renal, haematological, neurological, endocrine, gastrointestinal, or pulmonary diseases.
- Current smokers; former smokers with >10 pack-years history or who stopped smoking <6 months before Visit 1.
- Participation in another clinical study with a study intervention administered in the last 30 days or 5 half-lives (whichever is longer).
- Participants with known hypersensitivity to β_2-agonists, corticosteroids, or any component of the MDI.
- Any clinically relevant abnormal findings in physical examination, clinical chemistry, haematology, vital signs, or ECG.

ACQ, Asthma Control Questionnaire; ECG, electrocardiogram; FEV_1, forced expiratory volume in 1 second; ICS, inhaled corticosteroid; LABA, long-acting β_2-agonist; MDI, metered-dose inhaler.

SUPPLEMENTARY TABLE S2. Efficacy endpoints in the VATHOS study by regional approach (US, EU/ROW, and Japan submission approaches; Efficacy Set*)

	**US Submission approach**[a]	**EU/ROW submission approach**[a]	**Japan submission approach**[a]
**Comparisons**	BFF_A 320 versus BD at 24 weeks	BFF_A 320 versus BD over 24 weeks	BFF_A 320 versus BD over 12–24 weeks[b]
			BFF_A versus BFF_{DPI} (non-inferiority) over 12–24 weeks[b]
**Primary lung function endpoints**	Change from baseline in **FEV_1 $AUC_{0–3}$** at week 24	Change from baseline in **morning pre-dose trough FEV_1** over 24 weeks	Change from baseline in **morning pre-dose trough FEV_1** over 12–24 weeks
**Key secondary lung function endpoints**	Change from baseline in **morning pre-dose trough FEV_1** at Week 24	Change from baseline in **FEV_1 $AUC_{0–3}$** over 24 weeks	Change from baseline in **FEV_1 $AUC_{0–3}$** over 12–24 weeks
**Secondary Endpoints**	**Severe asthma exacerbations** (VATHOS only and pooled VATHOS and LITHOS)		
	Change from baseline in the mean number of puffs of **rescue medication use** (puffs/day) over 24 weeks	Change from baseline in the mean number of puffs of **rescue medication use** (puffs/day) over 24 weeks	Change from baseline in the mean number of puffs of **rescue medication use** (puffs/day) over 12–24 weeks
	Percentage of responders in **ACQ-7** (≥0.5 decrease equals response) at Week 24	Percentage of responders in **ACQ-7** (≥0.5 decrease equals response) over 24 weeks	Percentage of responders in **ACQ-7** (≥0.5 decrease equals response) over 12–24 weeks
	Percentage of responders in **ACQ-5** (≥0.5 decrease equals response) at Week 24	Percentage of responders in **ACQ-5** (≥0.5 decrease equals response) over 24 weeks	Percentage of responders in **ACQ-5** (≥0.5 decrease equals response) at over 12–24 weeks
	Percentage of responders in **AQLQ(s)+12** (≥0.5 increase equals response) at Week 24	Percentage of responders in **AQLQ(s)+12** (≥0.5 increase equals response) over 24 weeks	Percentage of responders in **AQLQ(s)+12** (≥0.5 increase equals response) at over 12–24 weeks
	**Onset of action** on Day 1: absolute change in FEV_1 at 5 minutes post-dose		

[a]Refers to submission strategy with respect to Health Authority responsible for reviewing marketing authorization, not the recruitment location/nationality of the participants.

bJapan was the only submission that had BFFDPI as a prespecified comparator, for trough FEV1 and FEV1 AUC0-3. Unless noted in table, comparisons are between BFFA and BD.

ACQ, Asthma Control Questionnaire; AQLQ(s)+12, Asthma Quality of Life Questionnaire for 12 years and older; BD, budesonide 320 μg via Aerosphere® MDI; BFFA 160, budesonide/formoterol fumarate dihydrate 160/10 μg via Aerosphere® MDI; BFFA 320, budesonide/formoterol fumarate dihydrate 320/10 μg via Aerosphere® MDI; BFFDPI, budesonide/formoterol fumarate dihydrate 320/9 μg via dry-powder inhaler; FEV1, forced expiratory volume in 1 second; MDI, metered-dose inhaler; N, number of subjects per treatment group; n, number of subjects in analysis; ROW, rest of world; US, United States.

SUPPLEMENTARY TABLE S3. Summary of efficacy endpoints, (Europe/ROW regional approach and Japan regional approach), Efficacy Set (used for superiority comparisons)

	**BFF_A 320**	**BD 320**
**Europe/ROW approach**		
**Primary endpoint**		
Change from baseline in morning pre-dose trough FEV_1 over 24 weeks, mL[a]	n=161	n=166
Estimate (SE)	106 (16.2)	25 (15.9)
LS mean difference (95% CI), p-value	80 (36, 125), p=0.0004[b]	
**Secondary endpoints included in the Type I error control procedure**		
Change from baseline in FEV_1 AUC_{0-3} over 24 weeks, mL[a]	n=161	n=166
Estimate (SE)	267 (16.2)	78 (15.9)
LS mean difference (95% CI), p-value	190 (145, 234), p<0.0001[b]	
Onset of action: change in FEV_1 at 5 minutes post-dose on Day 1, mL[a]	n=159	n=158
Estimate (SE)	184 (12.2)	41 (12.1)
LS mean difference (95% CI), p-value	144 (110, 177), p<0.0001[b]	
Percentage of 7-item ACQ responders over 24 weeks[a]	n=162	n=166
Responders, n (%)	111 (68.5)	99 (59.6)
Odds ratio (95% CI), p-value	1.549 (0.976, 2.460), p=0.0634	
Percentage of 5-item ACQ responders over 24 weeks[a]	n=162	n=166
Responders, n (%)	120 (74.1)	109 (65.7)
Odds ratio (95% CI), p-value	1.557 (0.959, 2.526), p=0.0731	
Percentage of AQLQ(s)+12 responders over 24 weeks[a]	n=153	n=162

Responders, n (%)	91 (59.5)		81 (50.0)	
Odds ratio (95% CI), p-value	1.732 (1.077, 2.784), p=0.0233[c]			
Change from baseline in mean puffs of rescue medication per day over 24 weeks[a]	n=157		n=163	
Estimate (SE)	-0.481 (0.0781)		-0.213 (0.0764)	
Mean change (95% CI), p-value	-0.268 (-0.482, -0.053), p=0.0146[c]			
**Japan regional approach**	**BFF_A 320**	**BD 320**	**BFF_A 320**	**BFF_{DPI}**
**Primary endpoints**				
Change from baseline in morning pre-dose trough FEV_1 over 12–24 weeks, mL[a]	n=159	n=164	–	–
Estimate (SE)	103 (17.9)	21 (17.6)	–	–
LS mean difference (95% CI), p-value	81 (32, 131), p=0.0012[b]		–	
NI change from baseline in morning pre-dose trough FEV_1 over 12–24 weeks, mL[d] (Per Protocol Set)[e]	–	–	n=108	n=117
Estimate (SE)	–	–	137 (20.8)	161 (20.1)
LS mean difference (95% CI), NI p-value	–		-24 (-81, 33), p=0.0047[b]	
**Secondary endpoints included in the Type I error control procedure**				
Change from baseline in FEV_1 AUC_{0-3} over 12–24 weeks, mL[a]	n=159	n=164	–	–
Estimate (SE)	255 (19.9)	72 (19.6)	–	–
LS mean difference (95% CI), p-value	183 (129, 238), p<0.0001[b]		–	
Onset of action: change in FEV_1 at 5 minutes post-dose on Day 1, mL[a]	n=159	n=158	–	–
Estimate (SE)	184 (12.2)	41 (12.1)	–	–
LS mean difference (95% CI), p-value	144 (110, 177), p<0.0001[b]		–	
Percentage of 7-item ACQ responders over 12–24 weeks[a]	n=160	n=164	–	–

Responders, n (%)	102 (63.8)	95 (57.9)	–	–
Odds ratio (95% CI), p-value	1.309 (0.832, 2.058), p=0.2445		–	
Percentage of 5-item ACQ responders over 12–24 weeks[a]	n=160	n=164	–	–
Responders, n (%)	109 (68.1)	105 (64.0)	–	–
Odds ratio (95% CI), p-value	1.217 (0.763, 1.941), p=0.4102		–	
Percentage of AQLQ(s)+12 responders over 12–24 weeks[a]	n=152	n=160	–	–
Responders, n (%)	83 (54.6)	77 (48.1)	–	–
Odds ratio (95% CI), p-value	1.512 (0.943, 2.423), p=0.0861		–	
Change from baseline in mean daily number of puffs of rescue medication over 12–24 weeks (inhalations per day)[a]	n=154	n=161	–	–
Estimate (SE)	-0.494 (0.0926)	-0.226 (0.0906)	–	–
Mean change (95% CI), p-value	-0.268 (-0.523, -0.014), p=0.0389[c]		–	
**Other secondary endpoint not in the Type I error control Procedure**				
NI change from baseline in FEV_1 $AUC_{0\text{-}3}$ over 12–24 weeks, mL[d] (Per Protocol Set[e])	–	–	n=108	n=117
Estimate (SE)	–	–	297 (21.7)	275 (20.9)
LS mean difference (95% CI), NI p-value	–		21 (-38, 81), p<0.0001	

[a]Primary strategy for handling ICEs.

[b]Significant per the Type I error control procedure.

[c]Nominally but not statistically significant due to the Type I error control procedure.

[d]Principal stratum estimand; the NI margin for morning pre-dose trough FEV_1: -100 mL; FEV_1 AUC_{0-3}: -125 mL.

[e]All participants in the Efficacy Set without an important protocol deviation that impacts efficacy at the date of randomisation

ACQ, Asthma Control Questionnaire; AQLQ(s)+12, Asthma Quality of Life Questionnaire for 12 years and older; AUC_{0-3}, area under curve from 0 to 3 hours; BD 320, budesonide 320 µg via MDI; BFF_A 160, budesonide/formoterol fumarate dihydrate 160/10 µg via Aerosphere® MDI; BFF_A 320, budesonide/formoterol fumarate dihydrate 320/10 µg via Aerosphere® MDI; BFF_{DPI}, budesonide/formoterol fumarate dihydrate 320/9 µg via dry-powder inhaler; CI, confidence interval; FEV_1, forced expiratory volume in 1 second; ICE, intercurrent event; LS, least squares; NI, non-inferiority; MDI, metered-dose inhaler; ROW, Rest of World; SE, standard error.

SUPPLEMENTARY TABLE S4. Descriptive statistics of lung function endpoints, Efficacy Set[a]

	**BFF_A 160**	**BFF_A 320**	**BD 320**	**BFF_{DPI}**
**FEV_1 $AUC_{0\text{-}3}$**				
Baseline				
n	87	161	166	159
Mean	2.313	2.164	2.136	2.100
SD	0.670	0.665	0.560	0.592
Week 24				
n	83	156	155	156
Mean	2.568	2.390	2.188	2.350
SD	0.799	0.733	0.600	0.724
Change from baseline				
n	83	155	160	155
Mean	0.273	0.225	0.071	0.247
SD	0.305	0.293	0.244	0.337
**Trough FEV_1**				
Baseline				
n	87	161	166	159
Mean	2.313	2.164	2.136	2.100
SD	0.670	0.665	0.560	0.592
Week 24				
n	84	156	161	156
Mean	2.420	2.252	2.166	2.241
SD	0.746	0.687	0.583	0.706
Change from baseline				
n	84	155	161	155
Mean	0.123	0.085	0.041	0.139
SD	0.269	0.254	0.245	0.338
**Onset of action**				
Baseline				
n	87	161	166	159
Mean	2.313	2.164	2.136	2.100
SD	0.670	0.665	0.560	0.592
5 minutes				
n	84	160	158	156
Mean	2.569	2.341	2.176	2.258
SD	0.771	0.706	0.591	0.635
Change from baseline				
n	84	159	158	156
Mean	0.240	0.177	0.039	0.160

SD	0.205	0.161	0.125	0.159

[a]All observed data were used unless participants discontinued prematurely in conjunction with asthma therapy or prohibited medication potentially impacting efficacy, in which case treatment failure was imputed. $AUC_{0–3}$, area under the curve from 0–3 hours; BFF_A, budesonide/formoterol fumarate dihydrate 320/10 µg via Aerosphere MDI; BFF_{DPI}, budesonide/formoterol fumarate dihydrate 320/9 µg via dry powder inhaler; FEV_1, forced expiratory volume in 1 second; MDI, metered dose inhaler; SD, standard deviation.

SUPPLEMENTARY TABLE S5. Effect of BFF doses (US regional approach), Efficacy Set

	**BFF_A 320 vs BFF_A 160**
Change from baseline in FEV_1 AUC_{0-3}, mL	
Estimated difference (95% CI), unadjusted p-value	**–36 (–109, 37), p=0.3340**
Change from baseline in morning pre-dose trough FEV_1, mL	
Estimated difference (95% CI), unadjusted p-value	**–26 (–97, 46), p=0.4780**
Percentage of 7-item ACQ responders	
Estimated odds ratio (95% CI), unadjusted p-value	**0.917 (0.509, 1.653), p=0.7740**
Percentage of responders 5-item ACQ	
Estimated odds ratio (95% CI), unadjusted p-value	**0.852 (0.457, 1.589), p=0.6148**
Percentage of AQLQ(s)+12 responders	
Estimated odds ratio (95% CI), unadjusted p-value	1.143 (0.642, 2.034), p=0.6505
Change from baseline in mean rescue medication use over 24 weeks, inhalations/day	
Estimated difference (95% CI), unadjusted p-value	–0.131 (–0.390, 0.127), p=0.3198
Severe exacerbations	
Estimated rate ratio (95% CI), unadjusted p-value	**1.40 (0.70, 2.82), p=0.3425**

Bold text numerically favours BFF_A 160; non-bold text numerically favours BFF_A 320.

ACQ, Asthma Control Questionnaire; AQLQ(s)+12, Asthma Quality of Life Questionnaire for 12 years and older; AUC_{0-3}, area under curve from 0 to 3 hours; BFF_A 160, budesonide/formoterol fumarate dihydrate 160/10 µg via Aerosphere® MDI; BFF_A 320, budesonide/formoterol fumarate dihydrate 320/10 µg via Aerosphere® MDI; CI, confidence interval; FEV_1, forced expiratory volume in 1 second; MDI, metered-dose inhaler; US, United States.

SUPPLEMENTARY TABLE S6. Percentage of Symptom-free Days Over 24 Weeks: While on Treatment Estimand (Efficacy Set)

				Comparison			
**Group**	**n**	**Estimate**	**SE**	**Estimate**	**SE**	**95% CI**	**p-value**
BFF_A 320 (N=162)	157	48.637	2.211	8.812	3.1052	(2.713, 14.912)	0.0047
BD 320 (N=166)	163	39.825	2.1770				

The analysis was performed using a repeated measures analysis of covariance. The model included treatment, the number of the relevant 4-week interval (interval 1 to 6), prior maintenance medication (ICS versus ICS-LABA), and the treatment-by-4-week interval interaction as categorical covariates, and baseline percentage of rescue-free days, baseline morning trough FEV_1, and percent albuterol reversibility were continuous covariates. Only participants with non-missing covariates were included in the analysis. A decrease in estimate for comparison favoured study drug.

BD, budesonide; BFF_A 160, budesonide/formoterol fumarate dihydrate 160/10 μg via Aerosphere® MDI; CI, confidence interval; FEV_1, forced expiratory volume in 1 second; ICS, inhaled corticosteroid; LABA, long-acting β_2-agonist; MDI, metered dose inhaler; N, number of participants in treatment group; n, number of participants in analysis; SE, standard error.

SUPPLEMENTARY TABLE S7. Summary of SAEs, Safety Set

**System Organ Class Preferred Term (MedDRA Version 27.1)**	**BFF_A 160 (N = 88)**		**BFF_A 320 (N = 163)**		**BD 320 (N = 168)**		**BFF_{DPI} (N = 162)**	
	**n**	**%**	**n**	**%**	**n**	**%**	**n**	**%**
Any SAE	3	3.4	3	1.8	1	0.6	7	4.3
Specific SAEs								
**Infections and infestations**	0	0.0	1	0.6	0	0.0	2	1.2
COVID-19 pneumonia	0	0.0	1	0.6	0	0.0	0	0.0
Diverticulitis	0	0.0	0	0.0	0	0.0	1	0.6
Localised infection	0	0.0	0	0.0	0	0.0	1	0.6
**Metabolism and nutrition disorders**	0	0.0	0	0.0	0	0.0	1	0.6
Decreased appetite	0	0.0	0	0.0	0	0.0	1	0.6
**Cardiac disorders**	0	0.0	0	0.0	1	0.6	0	0.0
Myocardial ischaemia	0	0.0	0	0.0	1	0.6	0	0.0
**Respiratory, thoracic and mediastinal disorders**	0	0.0	1	0.6	0	0.0	1	0.6
Asthma	0	0.0	1	0.6	0	0.0	1	0.6
**Gastrointestinal disorders**	2	2.3	0	0.0	1	0.6	1	0.6
Gastric ulcer	1	1.1	0	0.0	0	0.0	0	0.0
Gastritis	0	0.0	0	0.0	1	0.6	0	0.0
Inguinal hernia	1	1.1	0	0.0	0	0.0	0	0.0
Large intestine polyp	0	0.0	0	0.0	0	0.0	1	0.6
**Hepatobiliary disorders**	0	0.0	0	0.0	0	0.0	1	0.6

Cholecystitis acute	0	0.0	0	0.0	0	0.0	1	0.6
**Skin and subcutaneous tissue disorders**	0	0.0	1	0.6	0	0.0	0	0.0
Skin ulcer	0	0.0	1	0.6	0	0.0	0	0.0
**General disorders and administration site conditions**	0	0.0	0	0.0	0	0.0	1	0.6
Asthenia	0	0.0	0	0.0	0	0.0	1	0.6
**Injury, poisoning and procedural complications**	1	1.1	0	0.0	0	0.0	0	0.0
Clavicle fracture	1	1.1	0	0.0	0	0.0	0	0.0

SAEs with an onset date on or after the date of first dose of IP up to and including 1 day following the date of last IP dose. Participants with multiple occurrences were counted once per System Organ Class and Preferred Term regardless of the number of occurrences. BD 320, budesonide 320 µg via Aerosphere® MDI; BFF_A 160, budesonide/formoterol fumarate dihydrate 160/10 µg via Aerosphere® MDI; BFF_A 320, budesonide/formoterol fumarate dihydrate 320/10 µg via Aerosphere® MDI; BFF_{DPI}, budesonide/formoterol fumarate dihydrate 320/9 µg via dry-powder inhaler; IP, investigational product; MDI, metered-dose inhaler; MedDRA, Medical Dictionary for Regulatory Activities; N, number of participants per treatment group; n, number of participants in analysis; SAE, serious adverse event.

SUPPLEMENTARY FIGURE S1. Overview of Type I error Control MTPs for A) US regional approach; B) Europe/ROW regional approach; and C) Japan regional approach

A)

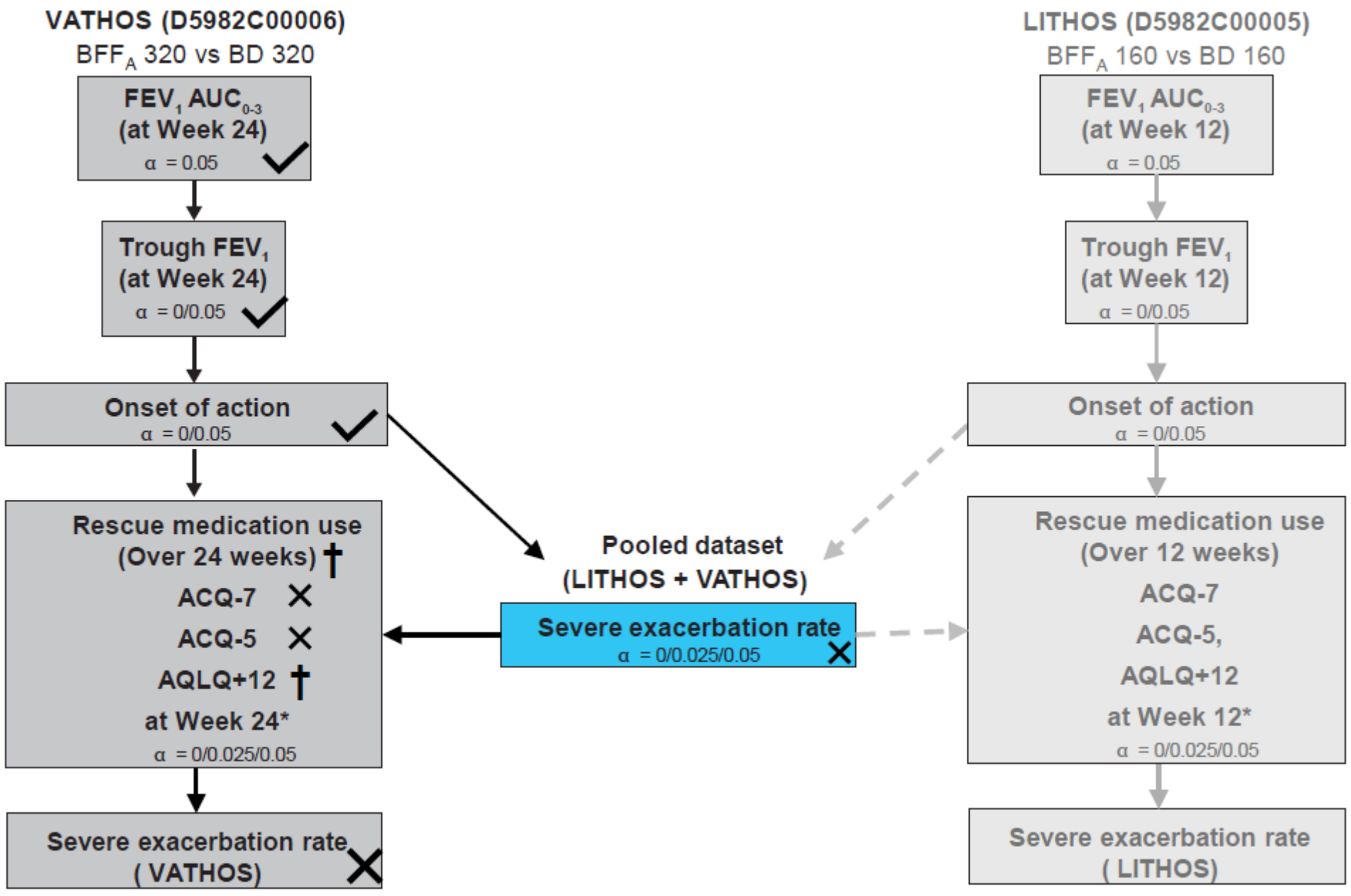

B)

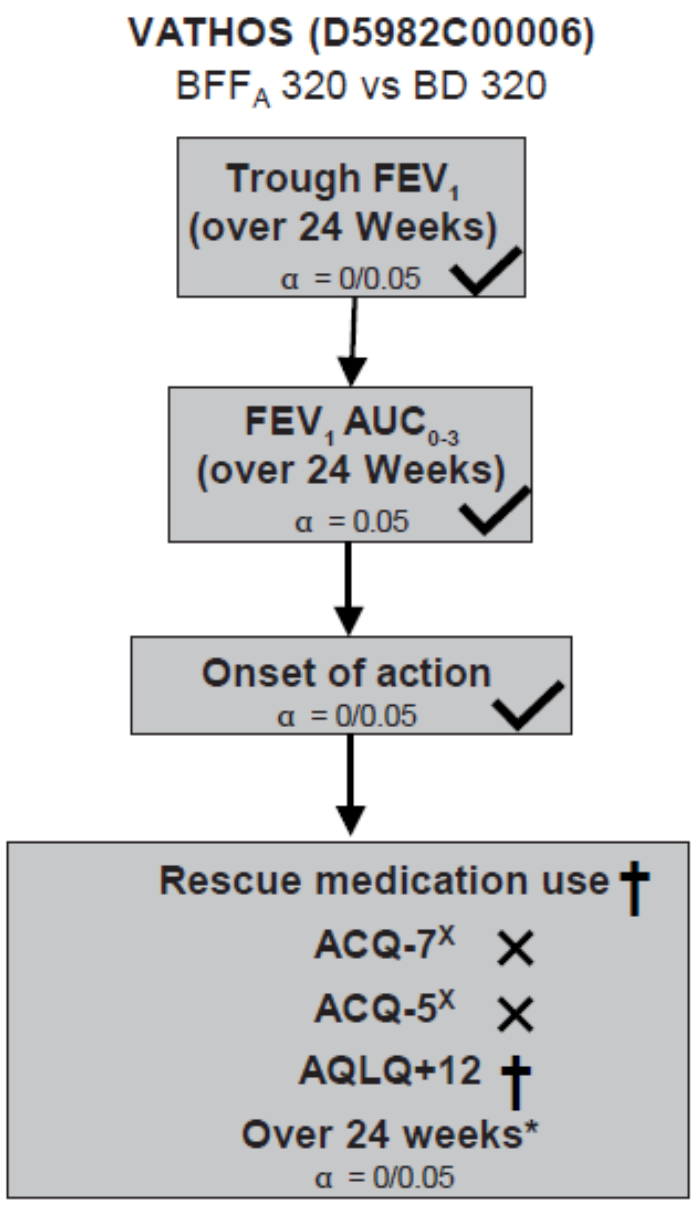

C)

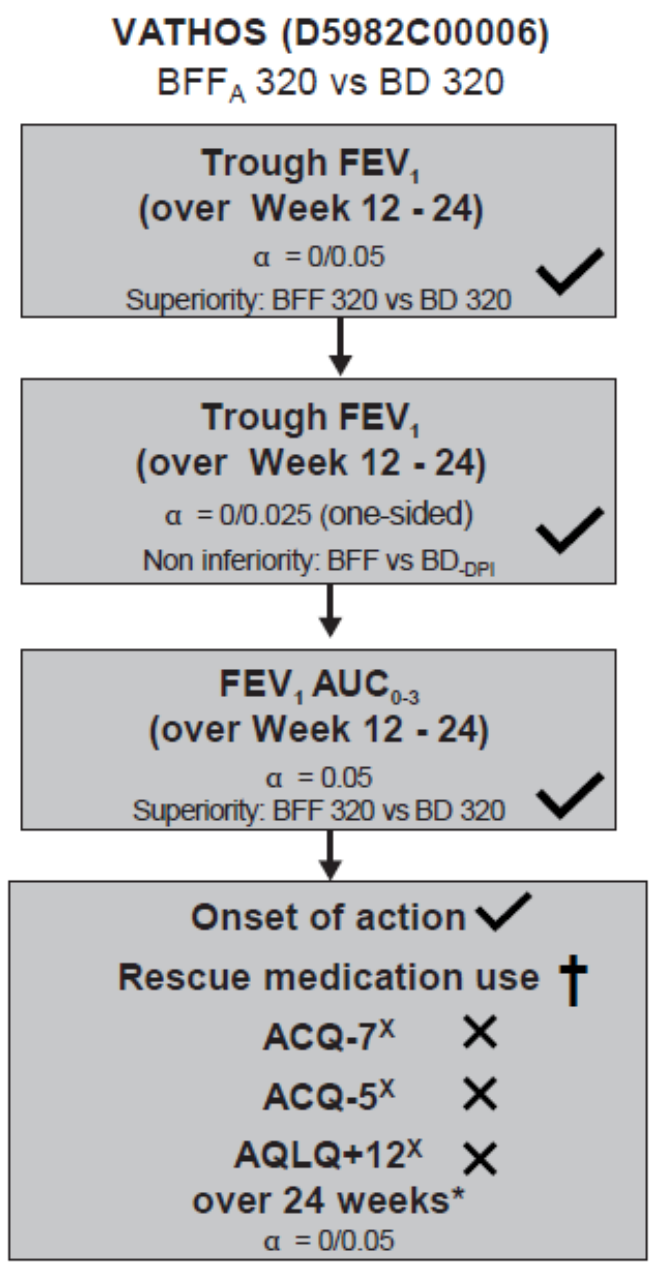

GCP violations were identified in 4 sites (in US and Japan). Data from affected participants were excluded from all analysis sets except the Screened Set.

*The test on change from baseline in onset of action, puffs of rescue medication and the percentage responder endpoints for ACQ-7, ACQ-5, AQLQ-12 will be strongly controlled using a Hochberg procedure.

†Denotes nominal significance due to placement in MTP: ✓ denotes statistical significance; X denotes no significance outside the multiple testing procedure.

ACQ, Asthma Control Questionnaire; AQLQ+12, Asthma Quality of Life Questionnaire for 12 years and older; AUC_{0-3}, area under curve 0 to 3 hours; BD, budesonide; BFF_A, budesonide/formoterol fumarate dihydrate 320/10 µg via Aerosphere MDI; BFF_{DPI}, budesonide/formoterol fumarate dihydrate 320/9 µg via dry powder inhaler; FEV_1, forced expiratory volume in 1 second; MTP, multiple testing procedures.

SUPPLEMENTARY FIGURE S2. Subgroup analyses of primary endpoints by regional approach: A) US (FEV_1 $AUC_{0\text{-}3}$ at Week 24); B) Europe/ROW (trough FEV_1 over 24 weeks); and C) Japan (trough FEV_1 over 12–24 weeks.)

A)

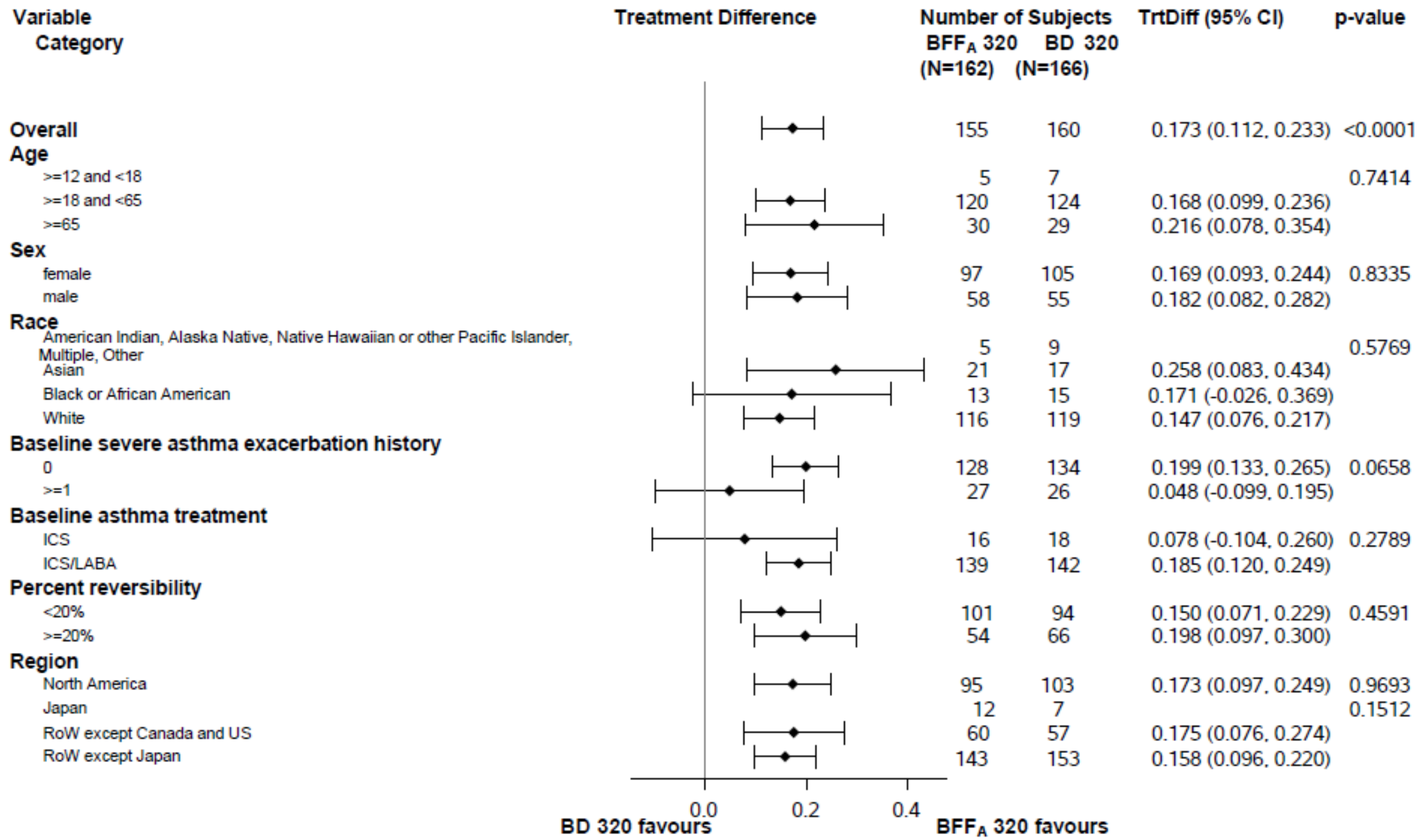

B)

Variable / Category	BFF$_A$ 320 (N=162)	BD 320 (N=166)	TrtDiff (95% CI)	p-value
**Overall**	161	166	0.080 (0.036, 0.125)	0.0004
**Age**				
>=12 and <18	6	7		0.3051
>=18 and <65	122	130	0.077 (0.026, 0.127)	
>=65	33	29	0.128 (0.027, 0.229)	
**Sex**				
female	100	108	0.088 (0.032, 0.145)	0.6539
male	61	58	0.067 (-0.007, 0.141)	
**Race**				
American Indian, Alaska Native, Native Hawaiian or other Pacific Islander, Multiple, Other	5	9		0.7329
Asian	21	18	0.110 (-0.019, 0.240)	
Black or African American	16	17	0.005 (-0.137, 0.147)	
White	119	122	0.080 (0.028, 0.132)	
**Baseline severe asthma exacerbation history**				
0	133	139	0.091 (0.042, 0.140)	0.3205
>=1	28	27	0.031 (-0.078, 0.139)	
**Baseline asthma treatment**				
ICS	17	20	-0.052 (-0.185, 0.081)	0.0378
ICS/LABA	144	146	0.097 (0.050, 0.145)	
**Percent reversibility**				
<20%	106	98	0.078 (0.019, 0.137)	0.8367
>=20%	55	68	0.068 (-0.008, 0.144)	
**Region**				
North America	100	107	0.063 (0.007, 0.119)	0.3032
Japan	12	8		0.8529
RoW except Canada and US	61	59	0.111 (0.038, 0.184)	
RoW except Japan	149	158	0.077 (0.032, 0.123)	

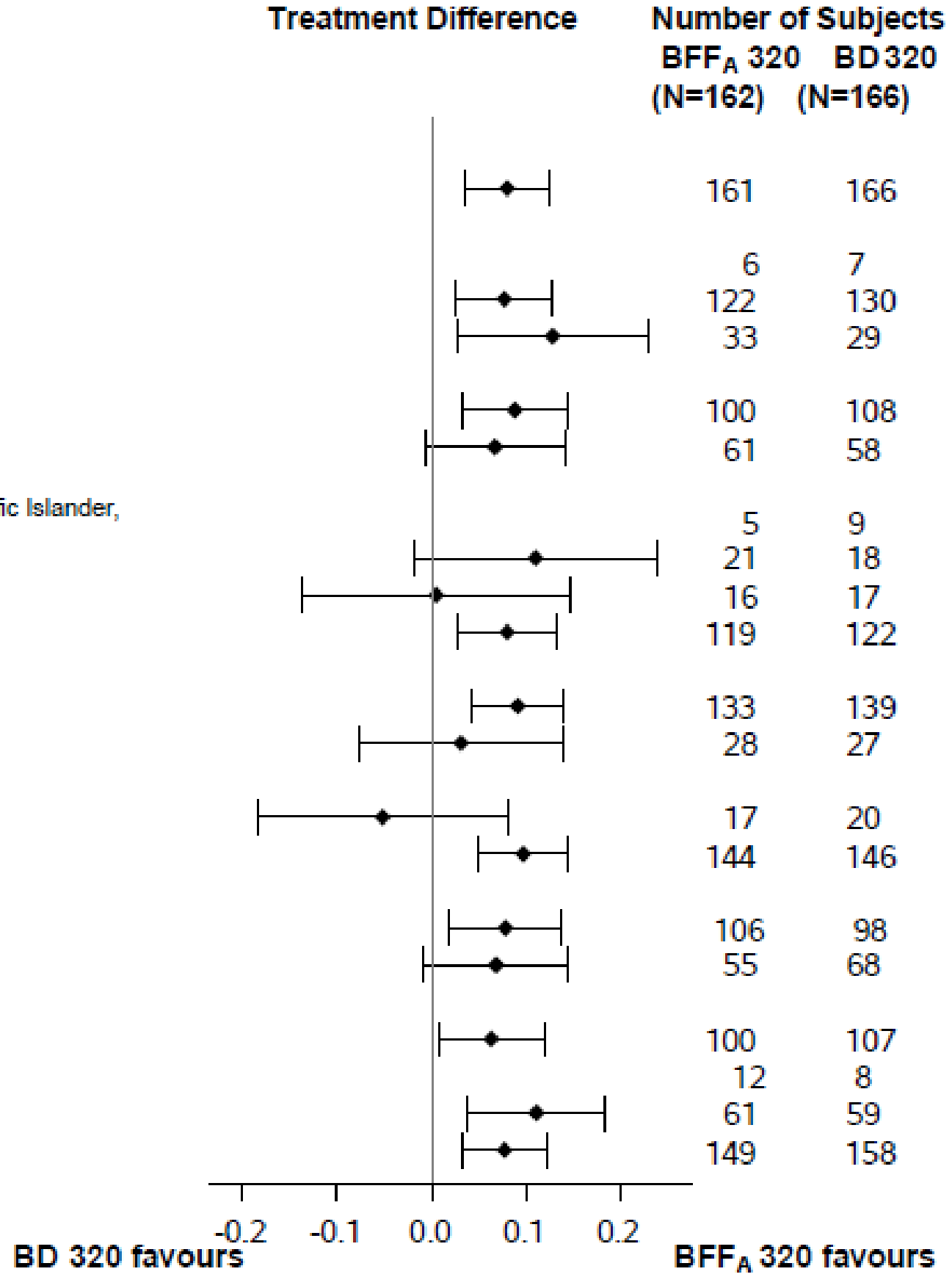

C)

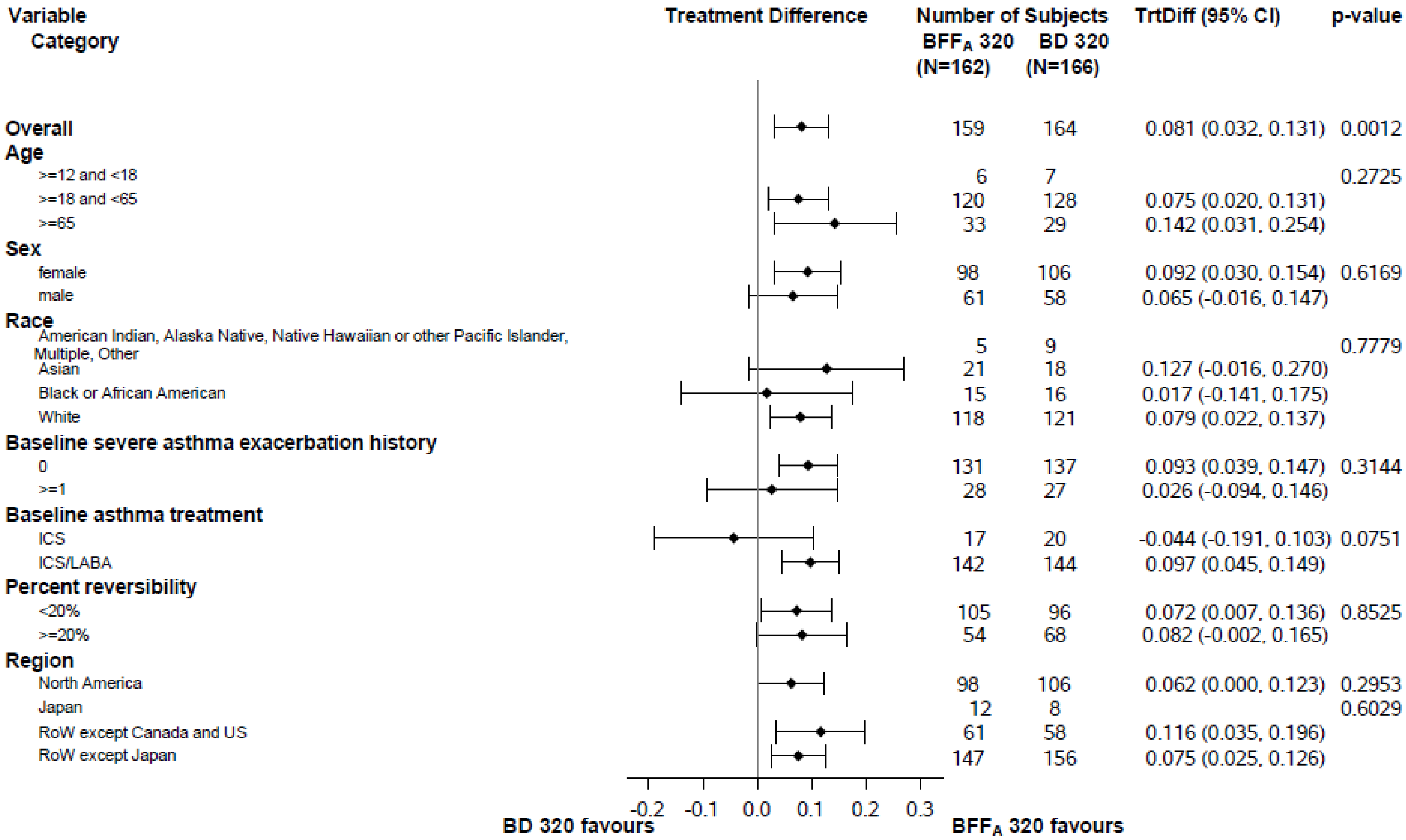

$AUC_{0\text{-}3}$, area under the curve from 0 to 3 hours; BD, budesonide; BFF, budesonide/formoterol fumarate dihydrate; CI, confidence interval; FEV_1, forced expiratory volume in one second; ICS, inhaled corticosteroid; LABA, long-acting β_2-agonist; ROW, Rest of World; US, United States.

SUPPLEMENTARY FIGURE S3. Change from baseline in onset of action at 5 minutes post-dose

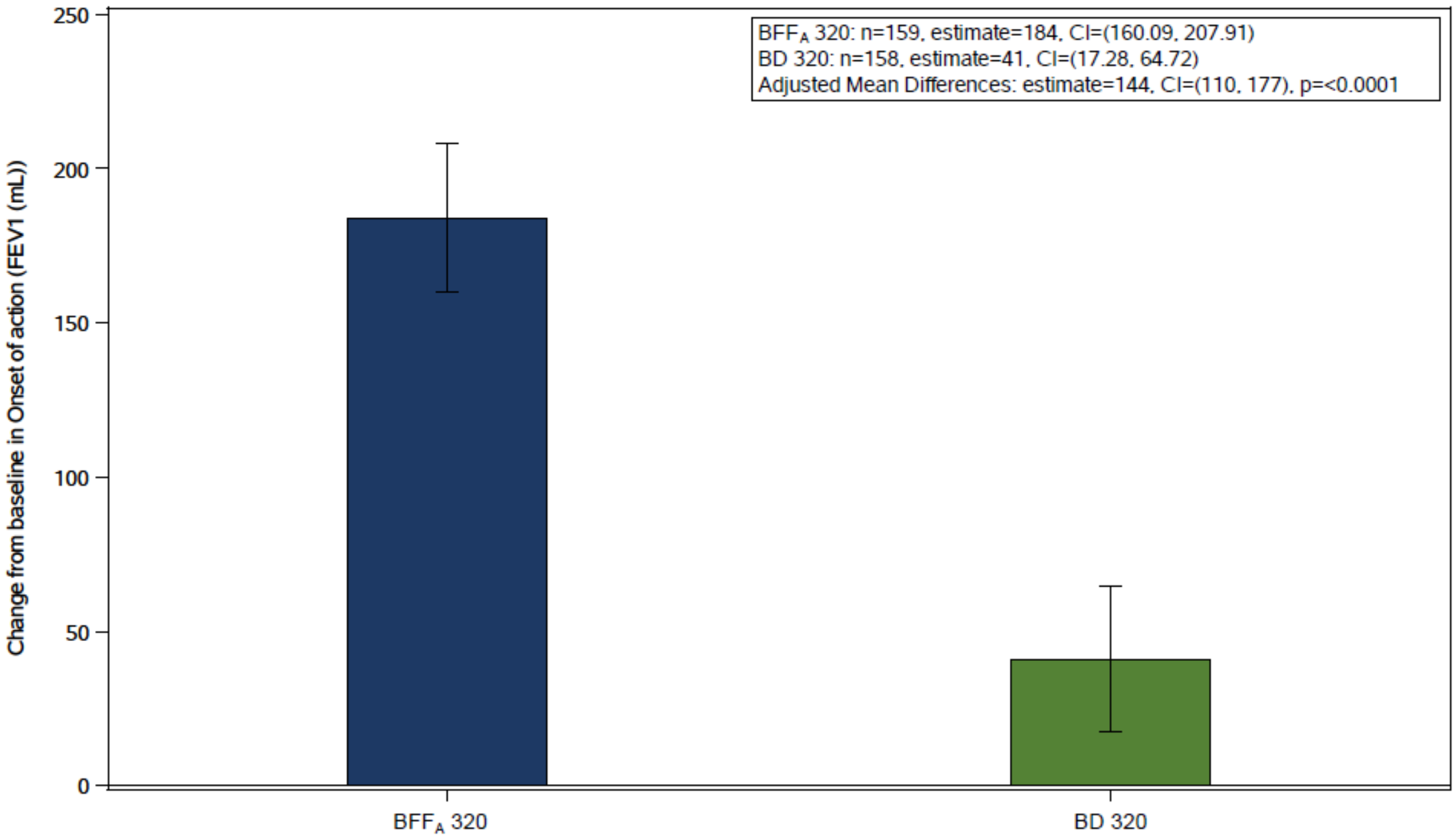

BD, budesonide via Aerosphere® MDI; BFF$_A$ 160, budesonide/formoterol fumarate dihydrate 160/10 μg via Aerosphere® MDI; CI, confidence interval; FEV_1, forced expiratory volume in one second.

SUPPLEMENTARY FIGURE S4. Generalized additive-model plot for A) change from baseline in FEV_1 $AUC_{0\text{-}3}$ and B) change from baseline in morning pre-dose trough FEV_1 at Week 24

A)

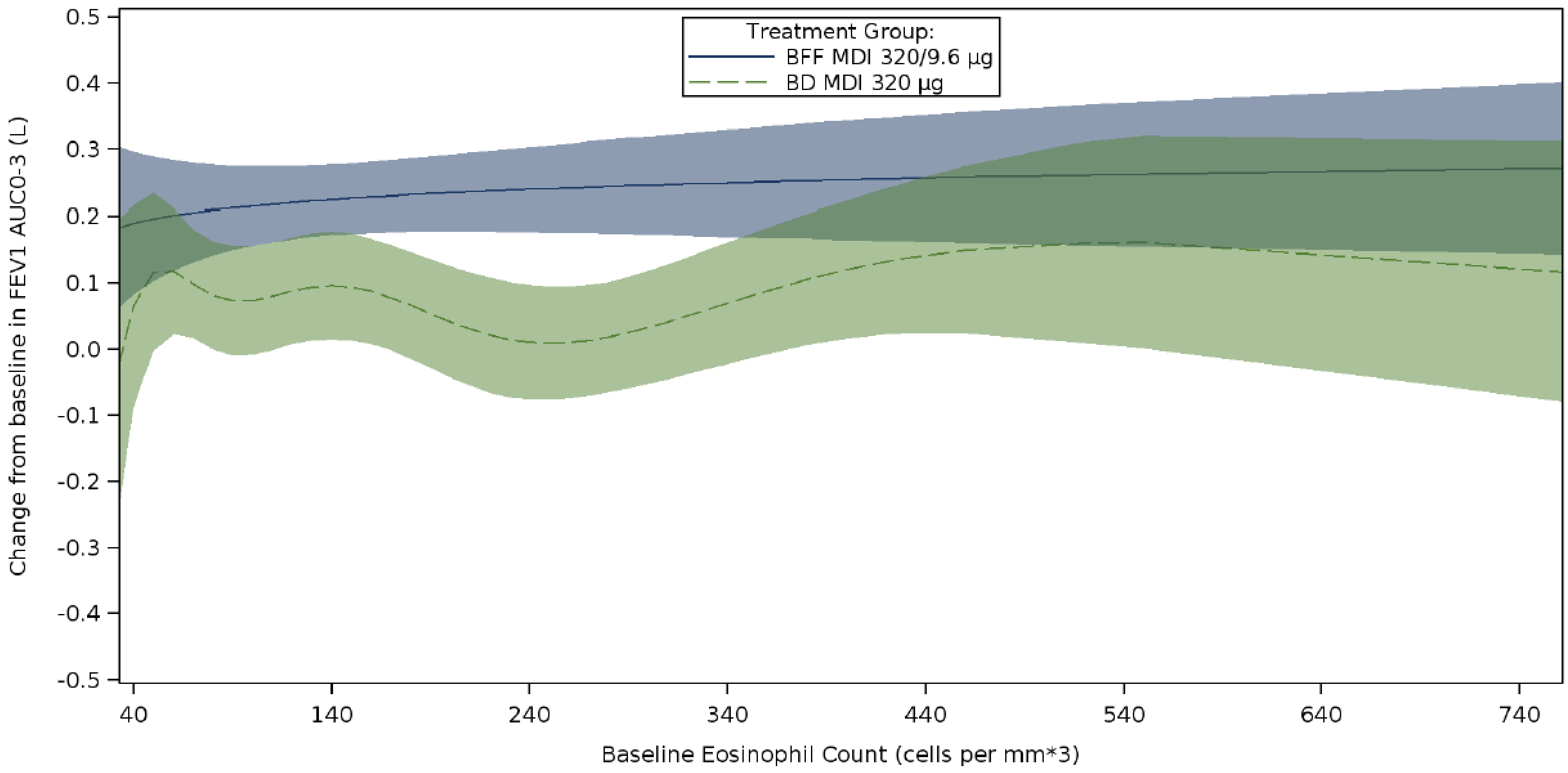

B)

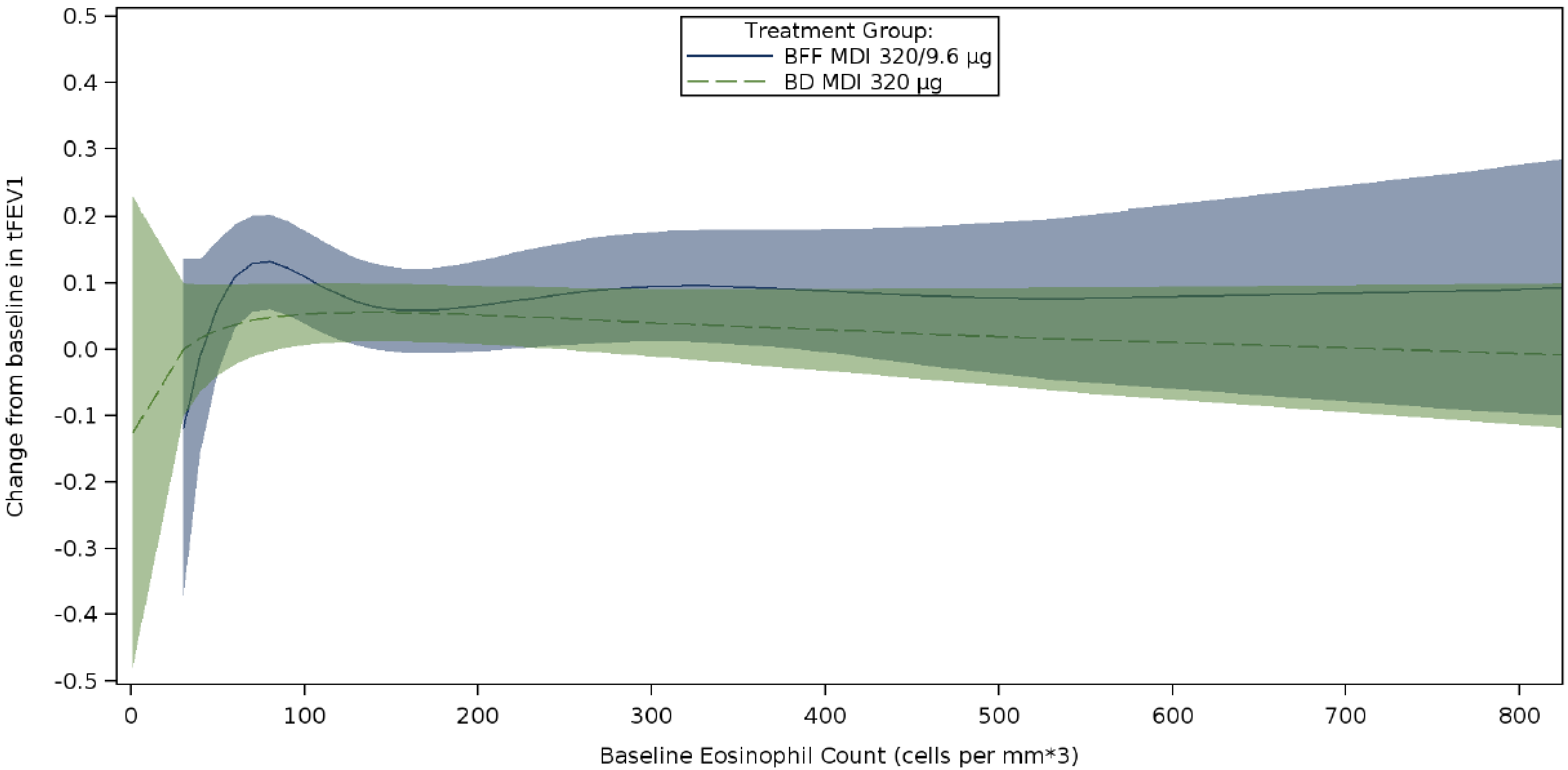

AUC_{0-3}, area under the curve from 0–3 hours; BD MDI 320 µg, budesonide 320 µg via Aerosphere® MDI; BFF 320 µg, budesonide/formoterol fumarate dihydrate 320/10 µg via Aerosphere® MDI; FEV_1, forced expiratory volume in 1 second; MDI, metered-dose inhaler.

SUPPLEMENTARY FIGURE S5. Change from baseline by timepoint in A) ACQ-5 and B) AQLQ(s)+12.

A)

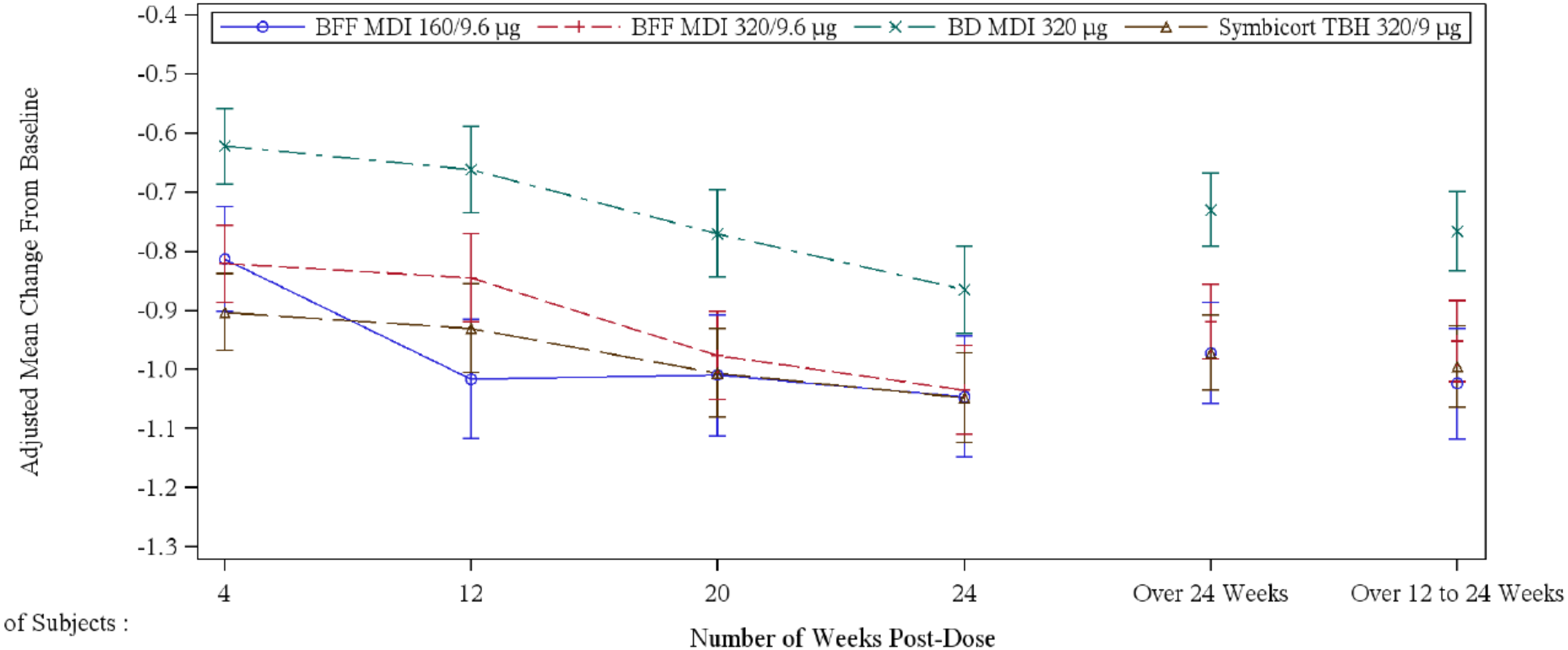

Number of Subjects :

	4	12	20	24	Over 24 Weeks	Over 12 to 24 Weeks
BFF MDI 160/9.6 µg	n=85	n=85	n=80	n=82	n=86	n=86
BFF MDI 320/9.6 µg	n=157	n=157	n=153	n=154	n=161	n=159
BD MDI 320 µg	n=165	n=162	n=154	n=161	n=166	n=164
Symbicort TBH 320/9 µg	n=156	n=157	n=155	n=153	n=157	n=157

B)

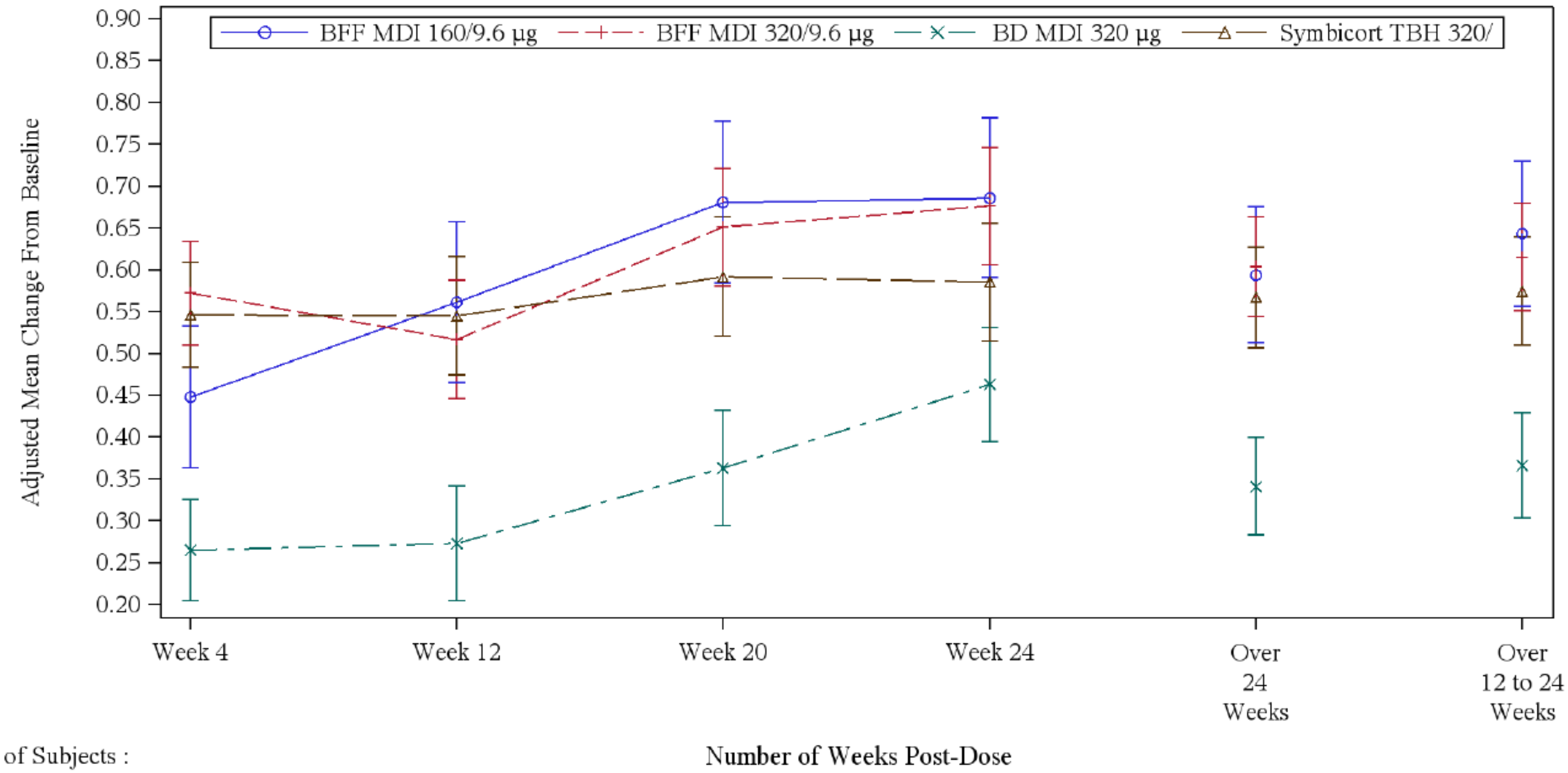

Number of Subjects :						
BFF MDI 160/9.6 µg	n=82	n=82	n=77	n=79	n=83	n=83
BFF MDI 320/9.6 µg	n=150	n=150	n=148	n=149	n=153	n=152
BD MDI 320 µg	n=160	n=158	n=151	n=157	n=162	n=160
Symbicort TBH 320/	n=149	n=150	n=148	n=147	n=150	n=150

Data are the adjusted means and standard error from analysis of the Efficacy Set. Only participants with non-missing covariates are included in the analysis. Baseline score for each ACQ analysis was defined as the last non-missing value (ACQ-5 or AQLQ(s)+12, respectively) before randomisation. The analysis was performed using a repeated

measures analysis of covariance model. The model included treatment, visit, prior maintenance medication (ICS versus ICS/LABA), and the treatment-by-visit interaction as categorical covariates and baseline trough FEV_1, percent albuterol reversibility, and ACQ-5 or AQLQ(s)+12 baseline score as continuous covariates.

ACQ, Asthma Control Questionnaire; AQLQ(s)+12, Asthma Quality of Life Questionnaire for 12 years and older; BD 320, budesonide 320 µg via Aerosphere® MDI; BFF_A 160, budesonide/formoterol fumarate dihydrate 160/10 µg via Aerosphere® MDI; BFF_A 320, budesonide/formoterol fumarate dihydrate 320/10 µg via Aerosphere® MDI; Symbicort TBH 320/9 µg (BFF_{DPI}), budesonide/formoterol fumarate dihydrate 320/9 µg via dry-powder inhaler; MDI, metered-dose inhaler.
